# Parameter-free hybrid RAO algorithm for reactive power optimization

**DOI:** 10.1038/s41598-026-58206-6

**Published:** 2026-06-18

**Authors:** Upma Singh, Saket Gupta, Mohd Zuhaib, Marwan Ahmad Abdullah Sufyan

**Affiliations:** 1 Department of Electronics and Communication Engineering, Maharaja Surajmal Institute of Technology, Delhi, 110058 India; 2 Department of Instrumentation and Control Engineering, Bharati Vidyapeeth’s College of Engineering, Delhi, 110063 India; 3 Department of Electrical Engineering, GLA University, Mathura, Uttar Pradesh India; 4 College of Engineering and Information Technology, Aljanad University of Science and Technology, Tiaz, Yemen

**Keywords:** Rao Algorithm, Optimization, Metaheuristics, Bibliometric Study, Hybrid Rao Algorithm, Engineering, Mathematics and computing

## Abstract

The Rao algorithms are a relatively new parameter-free optimisation technique that has grown in importance as an intelligent optimisation tool. Rao algorithms are effective algorithms that embody the idea of attracting toward a global optimum, illustrating the algorithm’s swarm intelligence and the survival of the fittest principle, like that of an evolutionary algorithm. However, it has found applications in other areas related to optimization, primarily in engineering practice, where it is analyzed and summarized based on the specifics of each situation. The absence of algorithm-specific parameters explains the wide applicability of Rao optimization. Although several studies have addressed these methods, limited effort has been made to provide a comprehensive summary of Rao algorithms. This work aims to provide up-to-date information on Rao algorithms, which will be useful for researchers in this field. Additionally, a hybrid Rao algorithm is developed to overcome the limitations of traditional Rao algorithms. The proposed algorithm was implemented in the IEEE 30-bus and IEEE 57 bus systems, to resolve the Optimal VAR Dispatch Problem, and to assess its effectiveness. Results show that hybrid Rao algorithm deliver higher quality solutions within a reasonable timeframe, outperforming other approaches stated in the recent literature. The paper ends with recommendations for future developments to improve the Rao algorithm’s performance.

## Introduction

Recent years have seen significant advancement in population-based Intelligent optimization algorithms, such as Dragonfly Algorithm (DA), Genetic Algorithm (GA), Bat Algorithm (BA), Whale Optimization Algorithm (WOA), Moth-Flame Optimization (MFO), Evolution strategy (ES), differential evolution (DE), Jellyfish Search Optimizer (JSO), Gorilla Troops Optimizer (GTO), Genetic Programming (GP), Grenade Explosion/Launcher Algorithm (GLA), shuffled frog leaping (SFL), Evolution programming (EP) and bacteria foraging optimization (BFO)^1–4^. While avoiding becoming caught in local optima, these algorithms are effective at finding global solutions to a range of optimization problems^5^. A significant number of components in the solution space are evaluated rapidly and simultaneously to attain this majority^6^. Additionally, they are suitable for complex optimization issues due to their generality and practicability of implementation^7^.

Meta-heuristic approaches typically place less focus on quality and search for approximations of solutions using heuristic notions that are independent of the specifics of the problem^8^. Because of their remarkable capacity to alter the optimisation problem search space, new work on metaheuristic-based methods has piqued the interest of AI researchers^9^. The metaheuristic-based method is a general-purpose optimization platform that may be tailored to a wide range of optimization issues^10^. It examines the problem search space using an iterative evolution technique and employs learning mechanisms that capitalize on gained knowledge^11^. The process of finding an adequate approximation solution for the given optimization issue is usually governed by certain algorithmic parameters^12^.

Approximate solution-focused techniques such as meta-heuristic and heuristic approaches don’t always need a mathematical demonstration of convergence. They frequently come into play when traditional optimization techniques fail to solve a problem satisfactorily^13^. While metaheuristic approaches to search and optimization issues are generally computationally faster than traditional approaches, they generally lack formal proof of convergence for the ideal solution^14^. Instead of searching for the best answer, they try to arrive at a close-to-ideal one. In a reasonable length of time, a feasible solution can be determined with the help of metaheuristics. Parameter tweaking is necessary for all metaheuristics, including population size, iteration count, and algorithm-based parameters. Furthermore, it can be necessary to modify some algorithm-specific factors to improve the efficiency of certain algorithms^15,16^..

Most population-based evolutionary algorithms need controlling parameters that are specific to the algorithm. PSO uses topology, coefficients, and learning rate; the genetic algorithm, for instance, uses crossover probability and mutation probability; Artificial bee colonies use crossover rates, the number of different bee species, and self-adaptive selection operators; harmony search algorithms use pitch adjusting rates, harmony memory size, and harmony memory considering rates. Finding the right settings for each algorithm’s parameters can improve the performance of the previously mentioned techniques^17,18^.

In 2020, Professor Rao introduced the Rao algorithms, which are metaphor-free optimization techniques^19^. The best and worst results from each iteration are combined with random interactions among possible solutions by the Rao Algorithms to rapidly determine the optimal solution. The algorithm requires only two standard parameters: population size and the maximum number of function evaluations, both of which are easy to adjust. It eliminates several parameters present in earlier metaphor-based algorithms, such as probability, intensity, and cohesion factors, which are often difficult to tune accurately^20^.

The paper makes the following contributions:

1. This study aims to produce a bibliometric assessment, that involves evaluating a thorough examination of the context and the latest developments in literature concerning the Rao algorithms. This paper also offers suggestions and serves as an inspiration for further research.
2. A comprehensive and bibliometric analysis of the Rao algorithms are provided in this work, with reference to the most recent research conducted in 2019 and 2024.
3. A new hybrid Rao algorithm that combines the adaptable elements of the Rao-1, Rao-2, and Rao-3 techniques has been created. The slow convergence to the near-global optimum and premature convergence issues are two drawbacks of the conventional Rao algorithms that the suggested hybrid method can overcome.
4. Throughout the search phase, the HRA technique provides flexibility to balance the algorithm’s capacity for both exploration and exploitation. The ORPD problem is solved for the first time using the suggested HRA techniques, which are highly effective at handling complicated restricted optimization problems.
5. The efficacy of the recommended hybrid approach has been tested on well-known mathematics problems as well as power system optimization challenges. Additionally, to evaluate the hybrid Rao algorithm’s effectiveness practically and its performance is compared to other stated meta-heuristic methods.
6. A comprehensive performance evaluation through multiple independent runs and statistical analysis (Friedman rank test) to ensure the robustness and reliability of the results obtained.

The rest of the paper is structured as follows: An overview of the principles of metaheuristic approaches is presented in Section II. The concepts underpinning Rao’s structure and its modifications are briefly explained in Section III. The proposed hybrid Rao algorithm is described in Section IV. The outcomes of the simulation and discussions are given in Section V, which follows. In Section VI, statistical analyses are covered. In Section VII discussions are done on the findings. The survey is concluded in Section VIII.

## Meta-heuristics overview

Most meta-heuristic techniques try to imitate physical, biological, or other natural principles by employing a range of operators. Exploration and exploitation are two key ideas influencing how a meta-heuristic method behaves^21^. The metaheuristic becomes a global search feature in the first instance, which is related to the Exploration operator’s capacity to diversify solutions in the search area^22^. However, “exploitation” refers to the capacity of local search and to the way operators use information gleaned from solutions to focus their search.

**Fig. 1:**
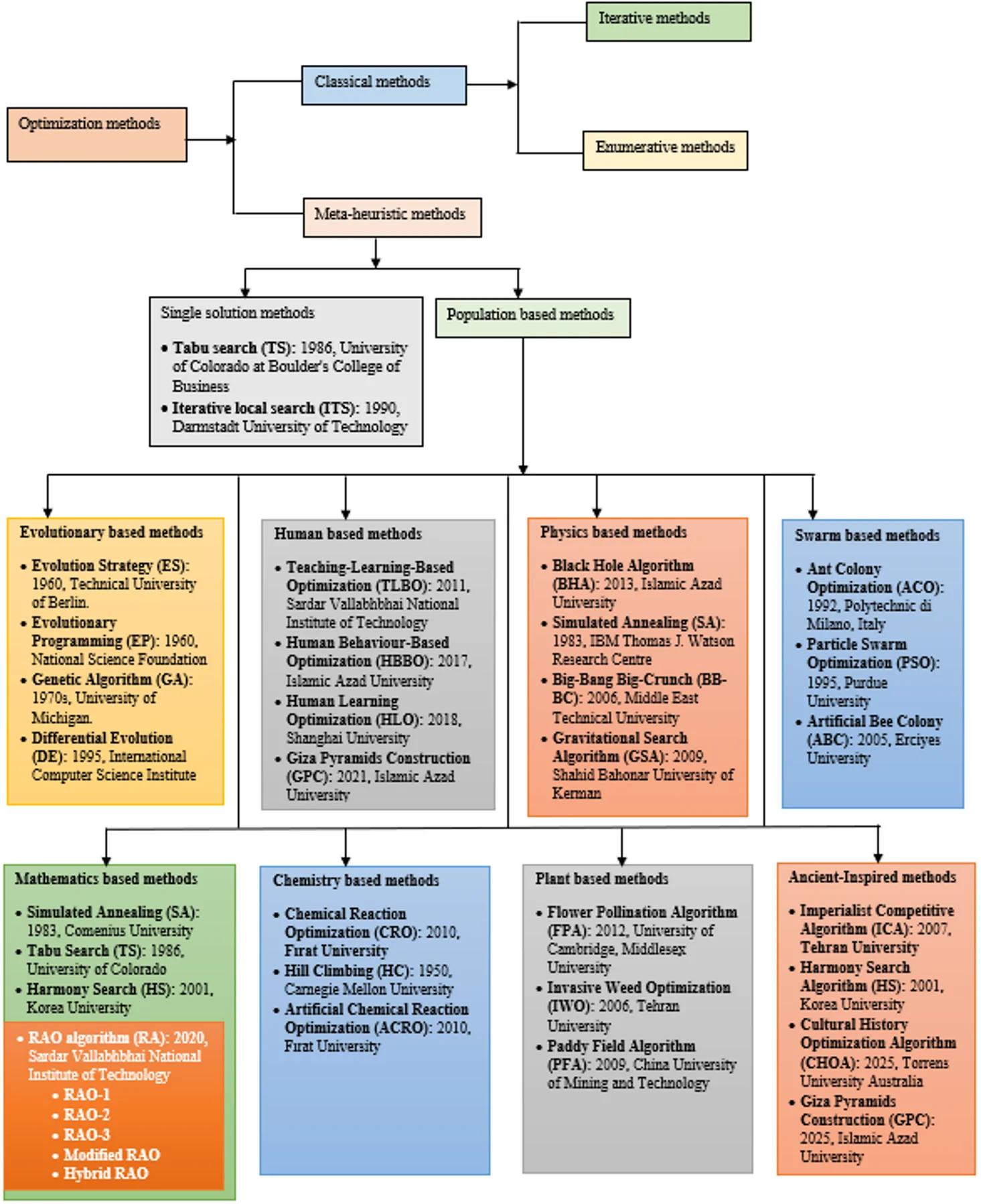
Classification of optimization algorithms.

Typically, metaheuristic-based algorithms are classified into several groups. They are population-based vs. single-point search, memory-based vs. memory-less; nature-based vs. non-nature-based; dynamic vs. static objective function. The scientific community has recently classified these algorithms based on inspiration: human-based, swarm intelligence, plant-based, chemical-based, evolutionary algorithms (EAs), mathematics-based, plant-based, and ancient-inspired algorithms^23,24^. Figure 1 generally depicts the general categorization of optimization approaches.

Holland and his pupils created a classic evolutionary algorithm called the genetic algorithm (GA) to resemble the biological concepts of survival of the fittest. It is one of the many meta-heuristic optimization techniques that have been successfully used over the past 20 years in a variety of optimization issues^25^. Natural evolutionary characteristics including genetic mutation, reproduction, and survival of the fittest serve as inspiration for EAs^26^. Natural selection, mutation, and recombination all contribute to the evolution of the initial population. Examples of distinct EAs are GP, GA, and EP^27^, Biogeography-Based Optimizer (BBO)^28^. Some examples include Probability-based Incremental Learning (PBIL)^29^, Evolution Strategies (ES)^30^, Differential.

Evolution (DE)^31^, etc. The second category of metaheuristic-based algorithms is swarm intelligence (SI). This family of algorithms is inspired by how swarms of animals work together to communicate, stay alive, and obey the swarm leader. The oldest SI approaches are Particle Swarm Optimisation (PSO)^32^ and Ant Colony Optimisation (ACO)^33^. The JAYA algorithm^34^, Honey Badger Algorithm (HBA)^35^, War strategy optimization algorithm^36^, Meerkat Algorithm (MA)^37^, Owl Optimisation Algorithm (OOA)^38^, Cheetah Based Optimisation Algorithm (CBOA)^39^, Harris Hawk Optimizer (HHO)^40^, Moth-fame optimization algorithm (MFO)^41^,, Bat algorithm (BA)^42^, Flower pollination algorithm (FPA)^43^, Grey Wolf Optimizer (GWO)^44^, Artificial Bee Colony (ABC)^45^, and Firefy algorithm (FA)^46^ are few of the approaches that are mentioned in the remainder of SI.

Metaheuristic algorithms with a physical foundation bear similarity to the physical mechanisms discovered in the cosmos. Physically based metaheuristics include the following: Artificial Chemical Reaction Optimisation Algorithm (ACROA) (Alatas 2011)^47^, Central force optimization (CFO)^48^, Archimedes Optimisation Algorithm (AOA)^49^, Thermal exchange optimization (TEO)^50^, Gases Brownian Motion Optimisation (GBMO)^51^, Multi-verse optimizer (MVO)^52^, Water evaporation optimization (WEO)^53^, Big Bang-Big Crunch (BBBC)^54^, Electromagnetic field optimization (EFO)^55^, and Gravitational search algorithm (GSA)^56^.

Particularly in the past ten years, metaheuristic algorithms have become an effective mechanism and complementary tool for more widespread applications^57^. Additionally, the “No Free Lunch (NFL)” theorem^58^, which holds that no one optimization strategy is best for all problems related to optimization, pushes researchers to constantly create new optimization algorithms to address difficult constraint optimization issues in a range of engineering and scientific domains, including ORPD problems.

## Real-world applications of rao algorithms

### History of Rao’s number of applications

**Fig. 2:**
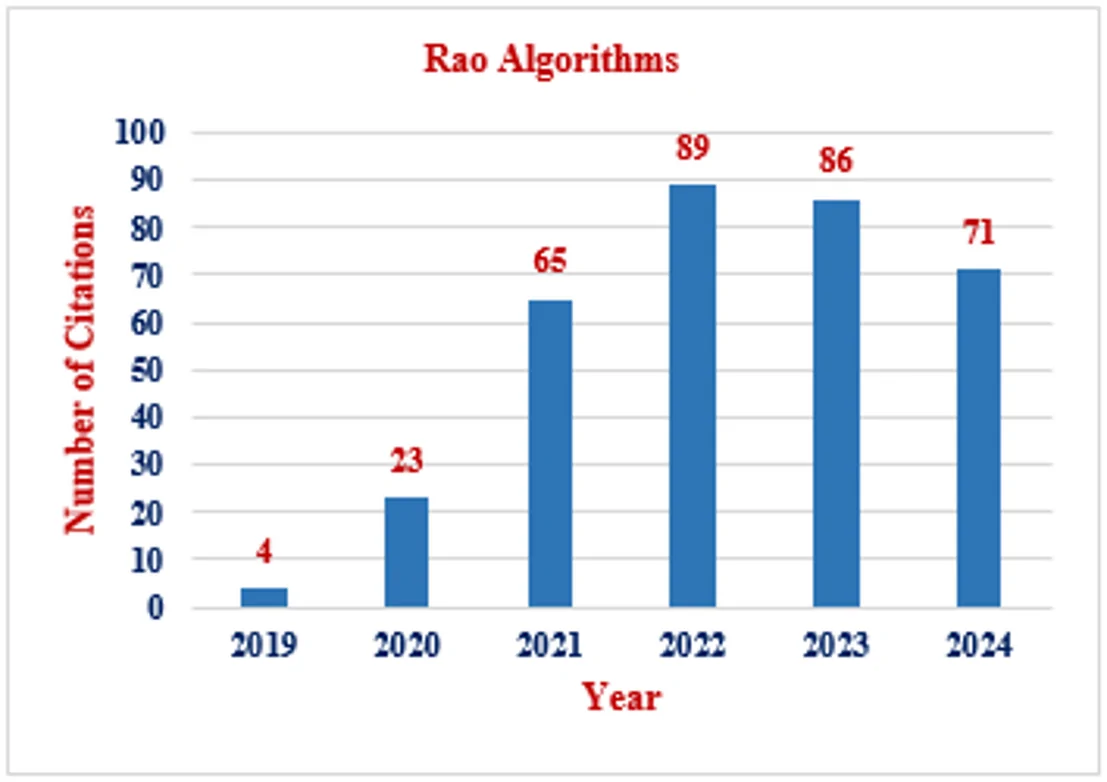
An outline of Rao’s publications during the years (Google Scholar: 19-11-2024).

This section provides many viewpoints on how the Rao algorithm has evolved. The Rao algorithm was initially developed in 2019 and released^59,60^. Since then, the Rao algorithm has experienced a sharp rise in popularity. As illustrated in Fig. 2, it has been published with numerous distinguished publishers, including Science Direct, Taylor & Francis, Hindawi, MDPI, Inderscience, IGI, IET Digital Library, IEEE Explorer, Springer-Link, Wiley, and many more, in respectable journals, conferences, and book chapters.

A detailed review of Rao algorithms is structured into six sections, encompassing different paradigms such as the applications of the classical Rao algorithm, Rao-1, Rao-2, Rao-3, and their modified and hybrid variants.

### Application of classical rao algorithm

**Fig. 3:**
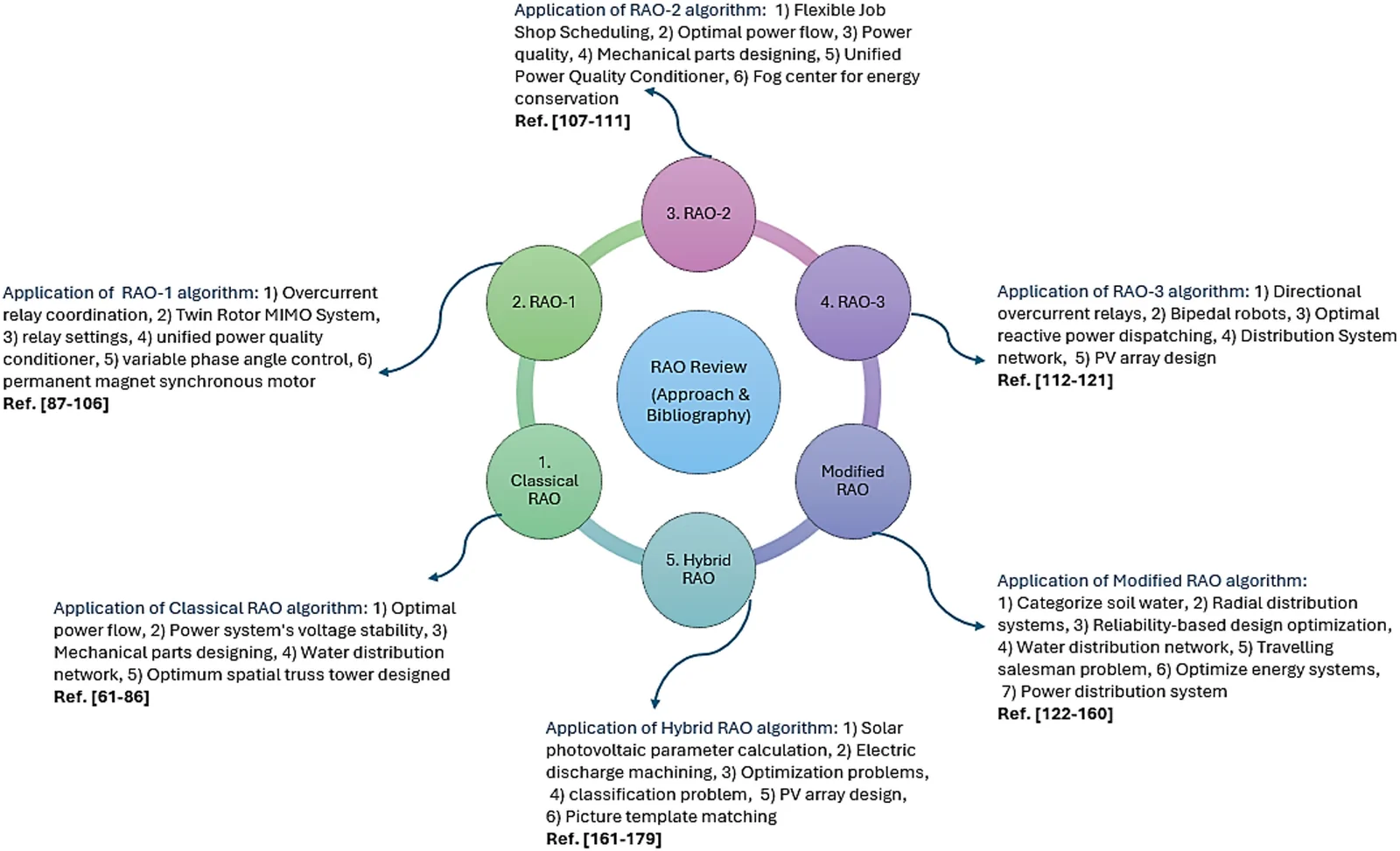
Circular display of the RAO’s suggested methodology and bibliography.

One of the most often cited repositories, Google Scholar, and Web of Science (WoS), were bibliometric data sources for this bibliographic study. “Rao Algorithm” or “Rao Optimisation” were the search terms entered into the query for both WoS and Google Scholar on Nov. 19, 2024. This analysis and comparison of the two databases cover publications from 2019, when the Rao was first published, to March 2024. The RAO’s proposed approach and bibliography are shown in a circular display in Fig. 3.

Saket Gupta et al. (2021)^61^ offer three logical, metaphor-free optimization strategies to address the OPF problem. Pushpendra Singh et al. created a novel approach for under voltage-based optimal load shedding in a power system in 2021. Three of Rao’s metaphor-free algorithms have been used to solve an optimization problem^62^. Jaya Lakshmi Ravipud et al. (2020) demonstrated the effectiveness of three newly created optimization methods, Rao-1, Rao-2, and Rao-3, in the construction of arrays of antennas. The main goal is to determine the ratio of ON to OFF components yields the lowest side lobe levels in thinning arrays with isotropic radiators. A comparison is made between the performance of the Rao-1, Rao-2, and Rao-3 algorithms with that of the hybrid Taguchi binary particle swarm optimization (HTBPSO), Biogeography-based optimization (BBO), Firefly algorithm (FA), Teaching-learning-based optimization (TLBO), and Rao-1, Rao-2, and Rao-3 algorithms produced lower side lobe level (SLL) antenna arrays compared to other optimization strategies^63^. Rao et al. (2020) investigated how well Rao algorithms worked for designing and optimizing specific mechanical system components. The designs generated by various optimization algorithms in previous studies are compared with the designs generated by Rao methods. The comparison of the results demonstrates that complex design optimization issues involving mechanical components can be solved with Rao methods^64^. The goal of Debasis Mohapatra et al. (2023) was to control and expedite the dissemination of knowledge. In this work, the inverse participation ratio (IPR) is utilized to evaluate the principal eigenvector’s (PEV) localization. We suggest three graph-based alternatives to Rao’s Algorithm as a step in this direction: Teaching-Learning-Based Optimisation (TLBO) Algorithm, Jaya technique for IPR Maximisation, and Rao’s Algorithm^65^. Using various Rao algorithm versions, R. V. Rao (2023) reported the design optimization of mechanical components^66^.

As water distribution utilized (WDNs) require expensive construction, the main goal of Priyanshu Jain et al. (2023) was network optimization to reduce network costs^67^. Additionally, utilizing two new optimization techniques, Rao-1 and Rao-2, Maksym Grzywiński et al. (2022)^68^ explain the integrated size and shape optimisation of spatial truss tower systems. According to Dulichand Jaraniya et al., a mix (AC-DC) charging station (CS) is connected to a solar panel array and an intelligent distribution grid. Using the Rao1, Rao2, and Rao3 algorithms, the optimal fitness values for the All previously developed Rao algorithms without metaphors are suggested in this paper as multi-objective variants. The proposed program handles many objectives at once by utilizing the crowding distance estimating approach and dominance principles. Two multi-attribute decision-making (MADM) techniques are used to identify unique solutions, including TOPSIS and R-method. Rao algorithms have been found to produce results that are competitive with those of earlier researchers who used different optimization techniques^70^. The goal of Jain, Priyanshu, et al. (2022) is to construct WDNs with optimal design by using a multi-objective Rao algorithm (MORao) that considers an existing modified resilience index (MRI)^71^. For second order plus dead-time (SOPDT) models, M. V. Srikanth et al. (2022) present the second order reduced linear Active Disturbance Rejection Control (ADRC). Finding a fair balance between monitoring and disturbance rejection performances is the goal when developing rules^72^.

To apply uncertainty theory to the multi-objective project selection problem, Kwon Ryong Hong et al. (2023) proposed a multi-objective mean-semivariance model that considers cooperation and reinvesting between projects with varying expenditure and functioning periods^73^. A multiple objectives mean-variance framework and related solution algorithms were examined by Xiaoxia, Huang, et al. (2022) in order to choose projects that consider synergy in an unpredictable environment^74^.

For processes that are modeled by First-order plus dead-time models (FOPDT), MV Srikanth et al. 2021^75^ have developed analytical tuning methods for RADRC. S. K. Refaay et al. (2023) presented Heterogeneous Wireless Sensor Networks (WSN) for Structure Optimization using Rao Algorithms^76^. Y.V. Deshpande et al. (2023) suggested Rao Algorithms for improving the performance of machining Inconel 625 milling^77^. U. Pati, and K.D Mistry (2023) proposed Rao Algorithms for reducing the distribution grid voltage imbalance problem^78^. Jitendra Singh Bhadoriya et al. (2023) suggested an Analysis of DSTATCOM and DG penetration in distribution systems under normal and critical loading conditions during COVID-19^79^. The ideal location for recuperation of energy and seepage lowering in water supply networks were suggested by Priyanshu Jain and Ruchi Khare (2023) using Rao algorithms^80^. R.V. Rao, and R.B. Pawar (2023) proposed Rao Algorithms for Mechanical Components Design^81^. L.V. Thinh, T.T. Huan, N.V. Long, (2024) suggested Rao Algorithm to solve the shortest path problem for urban traffic networks^82^. M. Yılmaz, T. Dede, R.V. Rao, (2023) proposed Rao algorithms for the Construction Scheduling Optimization problem^83^. E. Shrivastava, et al. (2023) recommend utilizing Rao algorithms for AI-based arc machining modeling and optimization^84^. C. O. Omeje and D. I. Angwe (2023) presented Rao Optimizers for reactive power dispatch problems^85^. S. Gade and R. Agrawal, (2023) proposed Rao Algorithm optimising a Converter’s VA Loading^86^. Tables 1, 2, 3, 4, 5 and 6.

**Table 1:** Applications of classical Rao algorithm.

Refs No.	Year	Method	Application	Contribution
^61^	2021	Rao	Optimal Power Flow	Optimal power flow solution achieved
^62^	2021	Rao	Voltage load shedding	Power system’s voltage stability obtained
^63^	2020	Rao	Antenna arrays	Concentric, planar, and linear circular antenna array synthesis achieved
^64^	2020	Rao	Spur Gear Train	Spur gear train’s ideal weight design issue resolved
^65^	2023	Rao	Localize information spread	Information that has spread over a network located
^66^	2023	Rao	Design of Mechanical Components	Mechanical parts designed
^67^	2023	Rao	Optimum Design of Pipe Network	Pipe network’s optimal design achieved
^68^	2022	Rao	Spatial truss towers	Optimum spatial truss tower designed
^69^	2022	Rao	Integration	The issue of incorporating charging stations for electric vehicles into a smart grid is addressed
^70^	2023	Rao	Solar Dish Stirling Heat Engine	A solar dish design engine that generates heat developed
^71^	2022	Rao	Design of water distribution networks	Water distribution networks’ resilience-based optimal design achieved
^72^	2022	Rao	Analytical tuning	Analytical tuning directions for the reduced ADRC of second order are obtained
^73^	2023	Rao	Project selection	Project selection using a multi-objective mean-semivariance model achieved
^74^	2022	Rao	Project selection	Project selection with an emphasis on collaboration achieved
^75^	2022	Rao	Shunt compensation and optimization	Nigerian 330 kV 34 bus power system’s shunt compensation obtained
^76^	2024	Rao	Wireless sensor networks	Structure of WSNs that are heterogeneous optimized
^77^	2023	Rao	Enhancing the machinability	Inconel 625 milling’s machinability improved
^78^	2023	Rao	Distribution networks	Three-phase power demands and lessen distribution networks’ voltage imbalance controlled
^79^	2023	Rao	Radial Distribution System	Mixed load models affected an imbalanced radial distribution system before and during the COVID-19 epidemic investigated
^80^	2023	Rao	Water distribution networks	Best site for water distribution networks’ energy recovery and leakage mitigation determined
^81^	2023	Rao	Design of Mechanical Components	Mechanical parts developed
^82^	2024	Rao	Building the Shortest Path Database	A database of the shortest routes in a city traffic system developed
^83^	2023	Rao	Construction Scheduling	Construction scheduling optimization achieved
^84^	2023	Rao	Modeling & optimization	Ti–6Al–4 V alloy arc machining simulated and improved
^85^	2023	Rao	Reactive Power Optimization	Distributed network’s reactive power optimization issue handled
^86^	2023	Rao	Loading Optimization	Converter’s VA loading to maximize the Unified Power Quality Conditioner’s (UPQC) optimised

### Application of RAO-1 Algorithm

Multiple buses overloading causes bus voltage violations and power outages in power system networks. The Nigerian 330-kV power network optimization task requires shunt compensation. ZO O. Jagun et al. (2022) introduce a new class of optimizer, Rao-1. Rao-1 is based on metaphor less programming and has quick convergence and simplicity^87^. Hagag Abdul Jabir et al. (2019) offer a straightforward optimization strategy for coordinating the electrical power system’s overcurrent relays. The directional overcurrent relays (DOCRs) are coordinated by a straightforward optimization method known as the Roa-1 algorithm^88^. Long Wang et al. (2020) presented a novel Rao-1-based photovoltaic (PV) cell parameter estimation technique^89^. The Rao-1 algorithm and variable phase angle control (PAC) are used by Swati Gade et al. (2022) to determine the ideal loading of the unified power quality conditioner (UPQC)^90^. Samia Batool et al. (2021), proposed a technique for creating an accurate, efficient, and optimal mechanism for over-current (OC) relay coordination in distribution networks^91^. Abdullah Shoukat et al. developed a method in 2021^92^ for computing the transmission line characteristics of a short line model employing measurements of voltages, currents, and power factors obtained at the line’s input and output. Sayani Sengupta et al. (2022) obtained more tuning parameters for the reported auto-tuner^93^ by using the Rao-1 optimization technique in a twin-rotor MIMO system (TRMS). For short-term solar irradiance forecasting, Zhongju Wang et al. (2022) developed a hybrid multi-modal ensemble learning model using sky photos and historical data. The Rao-1 method is chosen because of its ease of usage^94^. In this research, reactive power optimization of the Port Harcourt Electricity Distribution (PHED) was conducted by Crescent Onyebuchi Omeje et al. (2023), using a newly developed Rao-1 type optimization^95^. The Rao-1 method and particle swarm optimization (PSO) technique were used by Supriya, Naik, et al. (2023) to optimize the spoke-shaped internal permanent magnet synchronous motor (IPMSM) design parameters^96^. Similarly, several authors used Rao-1 for various purposes such as N. Agarwal et al.(2023)^97^, Z Wang et al. (2022)^98^, J Ku et al. (2022)^99^, L Bhukya et al. (2021)^100^, N Agarwal et al. (2022)^101^, AK Upadhyay et al. (2020)^102^. S. M. Reddy, M. Senthilkumar, and T.G. Sreekanth (2023) presented the Rao-1 algorithm for crack identification in cantilever beams^103^. N. Agarwal and M.R. Singh (2023) suggested the Rao − 1 algorithm for the optimization of TWR and SR of Superalloy^104^. S. Sengupta, C. Dey (2023) presented the Rao-1 algorithm for PID Controller Autotuning for MIMO Twin Rotor System^105^. N. Bharat, G. Akhil, and P.S.C. Bose (2023) proposed the Rao-1 algorithm for wear process parameters optimization^106^.

**Table 2:** Applications of Rao-1 algorithm.

Refs No.	Year	Method	Application	Contribution
^87^	2022	Rao-1	Shunt compensation and optimization	Best position and dimensions for shunt compensation components (such as reactors and capacitors) in a power system achieved
^88^	2019	Rao-1	Overcurrent Relays	The relay settings’ ideal values depending on the time dial setting (TDS) determined
^89^	2020	Rao-1	Photovoltaic cell	The problem of calculating unidentified parameters in a model of a solar power (PV) cell resolved
^90^	2022	Rao-1	UPQC	UPQC volt-ampere (VA) loading maximized
^91^	2021	Rao-1	Overcurrent relay	Overcurrent relay coordination problems resolved
^92^	2021	Rao-1	Short Transmission Line	Small transmission line’s parameters calculated
^93^	2023	Rao-1	PID Controller	TRMS with the best auto-tuned Proportional-Integral-Derivative controller developed
^94^	2022	Rao-1	Solar irradiance forecasting	A hybrid multi-modal ensemble learning model for forecasting solar irradiance in the short term was achieved
^95^	2023	Rao-1	Reactive Power Optimization	Distributed network’s reactive power optimization (RPO) problem resolved
^96^	2023	Rao-1	Spoke IPM motor	Spoke Interior Permanent Magnet (IPM) motor for efficiency improvement
^97^	2023	Rao-1	Optimization of TWR and SR	Tool Wear Rate (TWR) and Surface Roughness (SR), a superalloy, Al/9.6 B4C, were reduced
^98^	2022	Rao-1	Soil water retention	Soil water retention curve (SWRC) parameter estimate issue resolved
^99^	2022	Rao-1	Photovoltaic model	Photovoltaic (PV) model parameter estimated
^100^	2021	Rao-1	Solar PV system	Solar photovoltaic (PV) systems using maximum power point tracking (MPPT) achieved
^101^	2022	Rao-1	Optimization of material removal rate	Ti-6Al-4 V titanium alloy material removal rate (MRR) optimized
^102^	2020	Rao-1	Determination of closest saddle node	In a power system, the closest saddle node bifurcation point (CSNBP) is identified
^103^	2023	Rao-1	Crack detection in cantilever beams	Cantilever beam vibration-based fracture detection inverse problem resolved
^104^	2023	Rao-1	Optimization	Advanced optimization techniques to minimize two key metrics in the Electrical Discharge Machining (EDM) process were developed
^105^	2023	Rao-1	Twin Rotor MIMO System	An auto-tuned PID controller for a Twin Rotor MIMO System (TRMS) requires intelligent optimization designed
^106^	2023	Rao-1	Enhance Wear Characteristics	To optimize a new AA7178/nSiC metal matrix composite’s (MMC) wear property.

### Application of Rao-2 algorithm

Warid, Warid et al. (2022) provide the unique chaotic Rao-2 method as a fix for numerous OPF problems^107^. Rao-2 algorithm, a parameter-less and metaphor-less method, is used by Lalbihari Barik et al. (2021) to schedule the tasks in the fog center for energy conservation by meeting the QoS^108^. Dongsheng Yang et al. (2019) have applied three recently suggested meta-heuristic optimization algorithms—the Rao-2 method, MFO, and TLBO, to solve the FJSP to reduce the makespan^109^. A modified Rao-2 (MRao-2) technique was developed by Mohamed H. Hassan et al. (2021) to address the OPF problem in an electrical network that integrates renewable energy sources (RES)^110^. S. Gade et al.^128^ proposed the Rao-2 algorithm to mitigate the power quality problem using UPQC^111^.

**Table 3:** Applications of Rao-2 algorithm.

Refs No.	Year	Method	Application	Contribution
^107^	2022	Rao-2	OPF	The chaotic Rao-2 algorithm effectively improves the solution of OPF problems.
^108^	2021	Rao-2	Fog Assisted Cloud Environment	Fog-assisted cloud environment to address the problem of energy reduction obtained
^109^	2019	Rao-2	Flexible Job Shop Scheduling	FJS Problem resolved
^110^	2021	Rao-2	OPF	Incorporated RES into the OPF problem
^111^	2023	Rao-2	UPQC	UPQC (Unified Power Quality Conditioner) optimized

### Application of Rao-3 algorithm

Power systems protection devices are designed to isolate faults that arise in the protected zone as soon as possible from the remaining operational zone. Thus, Hagag Abdul Jabir et al. (2022) offer a simple optimization approach to synchronize the electrical power system’s directional overcurrent relays’ (DOCRs’) operations. The Roa-3 algorithm, a simple optimization technique, coordinates the DOCRs^112^.

Bipedal robots find it challenging to replicate human stride because it requires a sophisticated learning process involving the nervous system’s motor neurons coordinating with the body’s leg muscles. To attain optimal performance for the suggested model, Sravan Kumar Challa et al. (2022) have also determined the essential hyperparameter(s) through the application of the Rao-3 metaheuristic optimization technique (optimized-LSTM)^113^. The restricted non-linear optimal reactive power dispatching problem was tackled in a novel way by Mohamed H. Hassan et al. (2021) using the simple optimisation approach Rao-3^114^. While satisfying the planned demand for both power and heat, the main goal of combined heat and power economic dispatch, or CHPED, is to lower operational expenses. Three distinct algorithms, as proposed by Paramjeet Kaur et al. (2023), including the Jaya algorithm, the hybrid CHPED algorithm, and the Rao 3 algorithm, are utilized to optimize the dispatch operation. These algorithms contain various equality and inequality restrictions for generating units^115^. To enhance the task-scheduling approach, Ahmed Rabie Fayed et al. (2023) suggested an effective task-scheduling technique. The RAO-3 algorithm forms the foundation of the efficient task-scheduling method presented in this work for cloud computing systems^116^. Kalemci et al. (2020)^117^ provide an optimization algorithm for the construction of reinforced concrete retaining walls. Thanikanti Sudhakar Babu et al. (2020) employed three population-based optimization techniques to dynamically reorganize the PV array: The Rao optimization algorithm, the social mimic optimization algorithm (SMO), and the flow regime algorithm (FRA)^118^. Manjul Patel et al. evaluated the best-fused deposition modeling (FDM) situations using four algorithms—JAYA, RAO-3, PSO and bald eagle search optimization (BES) — To reduce both porosity and cylindricity error (CE). Gowdru Chandrashekarappa et al. (2021) conducted six case studies (sets of weights allotted for permeability and CE). The BES and RAO-3 algorithms immediately identified the optimal conditions^119^. A. K. Mohanty and P. S. Babu devised the fuzzy-based Rao-3 algorithm^120^ for distribution system reconfiguration. The Rao-3 algorithm was created by S. S. Halve, R. Arya, and A. Koshti to determine the optimal positions of various types of DG in a Distribution System network^121^.

**Table 4:** Applications of Rao-3 algorithm.

Refs No.	Year	Method	Application	Contribution
^112^	2022	Rao-3	Directional Overcurrent Relays	An optimization approach to synchronize directional overcurrent relays (DOCRs) operations using Rao-3 was achieved
^113^	2022	Rao-3	Human activity recognition	Applied Rao-3 to optimize hyperparameters of LSTM for gait learning (optimized-LSTM). obtained
^114^	2021	Rao-3	ORPD	Restricted non-linear optimal reactive power dispatch problem using Rao-3 tackled
^115^	2023	Rao-3	Combined heat and power economic dispatching	Dispatch operations under equality and inequality constraints achieved
^116^	2023	Rao-3	Task Scheduling	Task-scheduling technique for cloud computing systems developed
^117^	2020	Rao-3	Concrete cantilever retaining wall	An optimization algorithm for designing reinforced concrete retaining walls is developed
^118^	2020	Rao-3	Photovoltaic array	PV array reconfiguration achieved
^119^	2021	Rao-3	Accuracy and porosity of high impact polystyrene material	Showed BES and Rao-3 identified optimal conditions for porosity and CE.
^120^	2023	fuzzy-based Rao-3 algorithm	Electric Vehicle Charging	Fuzzy-based Rao-3 algorithm for the distribution system developed
^121^	2023	Rao-3	Radial distribution system	Optimal DG placement in distribution system networks is determined

### Application of modified Rao algorithm

To address single-, multi-, and many-objective optimization issues, R. Venkata Rao et al. (2021) presented the self-adaptive population Rao method^122^, which is an algorithm-specific parameter-free and metaphor-free method. To automatically categorize soil water content cycles, Z. Wang et al. (2021) suggested the hybrid SAX-VSM method (symbolic aggregate approximation and vector space model)^123^. According to R. V. Rao et al. (2021), the Rao-3, Rao-2, Rao-1, quasi-oppositional-based Rao-1 (QO Rao-1), self-adaptive multi-population Rao-1 (SAMP Rao-1), SAMP Rao-3, and SAMP Rao-2 algorithms can all be used to optimize the weight of a 10-bar truss design^124^. Using the Fuzzified RAO-3 algorithm, Ajit Kumar Mohanty et al. (2022) showed a fuzzy classified method for choosing the best-distributed generation (DG) sizes and locations, distribution static compensator (DSTATCOM), and electric vehicle charging station (EVCS) for radial distribution systems with 69 buses^125^. The sequential optimization and reliability assessment-double meta-heuristic (SORA-DM) framework by Ali Kaveh et al. (2022)^126^ is also used for the reliability-based design optimization (RBDO) of the frame structures. Three approaches were proposed by Ankit Kumar Nikum et al. (2021) to convert continuous Rao strategies to discrete equivalents. Two new mutation techniques are the basis for the other two strategies, while the swap operator that converts PSO into discrete PSO is the basis for one strategy. The symmetrical TSP problems are applied to the approaches, and the outcomes are contrasted with those of other cutting-edge algorithms, such as GA, the discrete bat algorithm (DBA), the ant colony algorithm, and the discrete cuckoo search (DCS). The results of the Rao algorithms are highly competitive when compared to other algorithms^127^.

The adaptive Rao approach (SAMP-Rao) was recommended by Abdelhady Ramadan et al. (2021) for solar cell parameter assessment^128^, more so than PV panels. For steel frame optimization in this study, Viet-Hung Truong et al. (2022) created a parameter-free metaheuristic built on the Enhanced Rao algorithms^129^. Using fusion-fission activity from the spider monkey optimization method^130^, Saurabh Pawar et al. (2022) updated the previous Rao equations to handle limited and unconstrained situations.

A workable modified Rao algorithm for boundary search has been demonstrated by Hoang-Anh Pham et al. (2022) to be a viable tool for optimizing the weight of truss structures^131^. The use of the SAMP-Rao3 algorithm in the difficult plate-fin heat exchanger (PFHE) design process was stressed by Ravipudi Venkata Rao et al. (2023)^132^. A unique colonial competitive optimizer (CCRAO) based on three modified RAO metaphor-free algorithms is introduced by Shahab S. Band et al. (2022)^133^. To improve the performance of the original Rao algorithm and get over its limitations—namely, the different features in exploitation and exploration—Mohamed Khamies et al. (2022)^134^ provide an improved optimization technique. A comprehensive description of Rao algorithms, Jaya algorithms, and their modified forms—such as the adaptive multi-team perturbation-guiding Jaya algorithm, the multi-team perturbation-guiding Jaya algorithm, the self-adaptive population Rao algorithm, and the elitist Rao algorithms—is given by Venkata Rao Ravipudi et al. (2022)^135^. The “Improved Rao (I-Rao) algorithm,” a successful and easily understood optimisation method, was presented by R. V. Rao et al. in 2023. The I-Rao algorithm comprises two stages: global exploration and local exploitation. The Rao algorithms’ search process can be more effectively exploited and their convergence time sped up during the local exploitation phase. The global exploration phase of the algorithm boosts its search efficiency and increases its ability to deviate from local optimum solutions^136^. The dimensional synthesis of four-bar path creation processes is given by Rao et al. (2023)^137^. Using the self-adaptive multi-population (SAMP) Rao algorithms, Ravipudi Venkata Rao et al. (2022) optimized the multi-objective design of nine spherical roller bearings (SRBs)^138^. The comprehensive learning Rao algorithm (CLRAO) is a novel metaheuristic technique that Patel Meet Prakashbhai et al. (2023) presented. This new version of the algorithm uses a thorough learning technique to enhance the global search capabilities of Rao algorithms and speed up convergence^139^. In contrast to metaphor-based algorithms, Yagmur Olmez’s (2023) multilevel picture thresholding approach uses a simple optimization strategy to decrease the complexity of the thresholding problem. More specifically, the Chaotic Enhanced Rao (CER) algorithms proposed in this study are developed using eight chaotic maps: Tent, Singer, Chebyshev, Circle Gauss, Sinusoidal, Sine, and Logistic. Furthermore, automatic threshold determination is used by the proposed CER algorithm instead of manual threshold decision^140^. Zinzuvadia Niketkumar Maheshkumar et al. (2022) introduce modified Rao algorithms, including self-adaptive multi-population elitist (SAMPE) Rao algorithms and chaotic Rao algorithms. The ten-bar truss structural optimization problems are solved using the algorithms given in^141^. To minimize the size of small truss tower structures while considering natural frequency as a constraint, Tayfun Dede et al. (2022)^142^ are investigating SAP-Rao. Forecasting PV system performance in applications in the industry is getting increasingly difficult due to the very nonlinear behavior of PV modules under different operating circumstances. To improve the efficiency of the so-called quasi-oppositional Rao-1 algorithm, a chaotic map is added, and the result is the quasi-oppositional logistic chaotic Rao-1 (QOLCR) algorithm. Quasi-oppositional logistic chaotic Rao-1 (QOLCR) algorithm^143^ is the name given to the algorithm by Mohamed Benghanem et al. (2023) when they incorporate a chaotic map to increase the algorithm’s efficacy. Jangkung, Raharjo, et al. (2022) employed the evolutionary Rao algorithm (ERA)^144^ to tackle IFSAR problems on Singapore Airlines flight datasets collected during the COVID-19 pandemic. R. Venkata Rao et al. (2020) proposed improved optimization methods based on self-adaptive multi-population^145^ to handle engineering design optimization difficulties. H. M. Sultan et al.^146^ proposed a chaotic Rao algorithm for chaotic Rao parameters estimation of proton exchange membrane fuel cells. C. Lin et al. recommended a multi-population Rao optimizer for monitoring the health of dams^147^. M. Ghasemi et al. (2023) presented a Fully Informed Search Algorithm which is based on Rao algorithms^148^. M. F. Tefek (2023) presented elite local search-based Rao algorithms^149^. R. V. Rao et al. (2023) presented Energy System Optimisation Using Elitist Rao Algorithms^150^. Similarly, other researchers used modified versions of Rao algorithms to solve different optimization issues in Science and Engineering: fuzzified RAO-3^151^, Chaotic Gaussian-Cauchy RAO (CGCRAO) algorithm^152^, Modified Mutation based Rao-3 Algorithm^153^, E-Rao^154^., GPU-Based based Rao techniques^155^, Discrete Rao Algorithm^156^, modified Rao’s algorithm^157^, and self-adaptive multi-population (SAMP) Rao algorithms^158^. G. Maksym, D. Tayfun and A. Barbaros (2023) proposed for optimal structure designs of steel grillage using self-adaptive population RAO algorithms^159^. S. Pravesjit et al. (2023) presented Improve Rao Algorithm for Optimization Problems^160^.

**Table 5:** Applications of modified Rao algorithm.

Refs No.	Year	Method	Application	Contribution
^122^	2021	Self-adaptive population Rao algorithm (SAPRA)	Bio-energy systems	Optimizing the functionality and design of specific bioenergy systems obtained
^123^	2021	Hybrid symbolic aggregate approximation (SAX) algorithm	Soil water content cycle classification	Automatically categorize cycles of soil water content using information from soil moisture sensors achieved
^124^	2021	SAMP Rao-algorithm.	Weight optimization of a truss structure	Truss structural weight optimization problem. solved
^125^	2022	Fuzzified RAO-3	Electric Vehicle Charging Stations	The best location for D-STATCOMs, DGs, and EV charging stations in a power distribution system has been achieved
^126^	2022	Rao-2, Rao-1, Shuffled shepherd optimization algorithms (SSOA)	Design optimization of the frame structures	Frame structures using the RBDO method achieved
^127^	2021	Novel discrete version of the Rao algorithms	Travelling Salesman Problem	Travelling Salesman Problem (TSP) solved
^128^	2023	Elitist Rao algorithms and the R-method	optimization of energy systems	Optimize energy systems. obtained
^129^	2021	SAMP Rao-algorithm.	Photovoltaic Cells	PV cell models’ unknown parameters calculated
^130^	2023	Fission Fusion Behavior-Based Rao Algorithm (FFBBRA)	Optimization problems	Algorithm’s equilibrium between exploitation and exploration, increasing its efficiency in resolving challenging optimization problem improved
^131^	2022	Modified Rao algorithm combined with a feasible boundary search method (FBSM)	Optimal truss sizing	Optimal truss sizing problem solved
^132^	2023	Self-Adaptive Multi-Population Rao-3	Plate-Fin Heat Exchanger	Plate-fin heat exchanger design optimized
^133^	2022	CCERA	Engineering design	The most effective way to address these engineering problems achieved
^134^	2022	Improved Rao algorithm	Nonlinear power system	Frequency stability of a nonlinear power system enhanced
^135^	2022	Elitist Rao algorithms and self-adaptive population Rao algorithm.	Optimization Problems	Multi-objective optimization problems resolved
^136^	2023	Improved Rao algorithm	Mechanical design optimization	Problems with optimization with a limited mechanical design resolved
^137^	2023	SAMP- Rao algorithm,	Dimensional synthesis of four-bar mechanisms	The four-bar mechanisms’ dimensional synthesis was carried out
^138^	2023	SAMP- Rao algorithm	Spherical Rolling Bearings	Rolling spherical bearings developed
^139^	2022	Comprehensive learning Rao algorithm (CLRAO)	Engineering optimization	Optimization problems in engineering handled
^140^	2022	Chaotically enhanced Rao algorithm	Image segmentation	Image segmentation achieved
^141^	2023	SAMP- Rao algorithm	Design of 3D Truss Tower	3D truss tower structure’s ideal weight design issue resolved
^142^	2022	SAMP- Rao algorithm	Truss Structures	Truss structural weight optimization problem resolved
^143^	2023	QOLCR algorithm	PV cells/modules	Performance of photovoltaic (PV) modules and cells enhanced
^144^	2022	Evolutionary Rao Algorithm	Flight Scheduling and Aircraft Routing	Optimal aircraft routing and integrated flight scheduling obtained
^145^	2020	SAMP- Rao algorithm	Engineering design optimization	Optimization of engineering designs achieved
^146^	2024	Chaotic Rao Optimization Algorithm	Fuel cell	Stacks of proton exchange membrane fuel cells (PEMFCs) simulated and analysed
^147^	2023	Multi-population Rao algorithm	Health Monitoring of Concrete Dam	For concrete dam health monitoring.
^148^	2023	Fully Informed Search Algorithm	Real-world problems	Real-world problems optimized
^149^	2022	ELSRao-1, ELSRao-2 and ELSRao-3.	Elite local search	Challenging and continuous optimization issues handled
^150^	2022	Elitist Rao algorithm	Energy Systems	Different energy systems’ designs. improved
^151^	2024	Fuzzified RAO-3	Electric vehicle charging stations	Best possible network reconfiguration of a power distribution system achieved
^152^	2023	Chaos-based Generalized Competitive Rao Algorithm (CGCRAO)	Permanent Magnet Synchronous Motor	Permanent Magnet Synchronous Motor’s (PMSM) parameters determined
^153^	2023	Modified Mutation-based Rao-3 Algorithm	Design Optimization of Surface Inset PMSM	Surface Inset Permanent Magnet Synchronous Motor’s (SPMSM) layout improved
^154^	2023	Enhanced Rao Algorithm (ERao)	Frame Structures	Frame structures developed
^155^	2023	Three Rao metaphor-less optimization algorithms	Large Systems of Nonlinear Equations	Large systems of nonlinear equations resolved
^156^	2023	Metaphor-less metaheuristic algorithm	Solving Combinatorial Optimization Problems	Combinatorial optimization issues resolved
^157^	2023	Opposition-Based Modified Rao’s Algorithm	OPF	Optimal Power Flow issue resolved
^158^	2023	self-adaptive multi-population (SAMP) Rao algorithms	Spherical Rolling Bearings	Spherical rolling bearings. designed
^159^	2023	Self-adaptive population Rao (SAP-Rao) algorithms	Steel grillage structures	Steel grillage structures optimized
^160^	2023	Whale Optimization Algorithm (WOA) with the Rao algorithm.	Optimization Problems	Improvement for problems with optimization achieved

### Application of HYBRID Rao algorithm

A discrete Rao-combined artificial bee colony (ABC) method was proposed by Kwon Ryong Hong et al. (2023) to construct inspection paths in radioactive environments with barriers that demand the least amount of exposure^161^. A hybrid Rao-Nelder-Mead (Rao-NM) algorithm for comparing image templates was proposed by Xinran Liuet et al. in 2021. The developed approach serially incorporates the NM and Rao-1 algorithms^162^. Sakkayaphop Pravesjit et al. developed a hybrid PSO and Rao method to categorize breast cancer The velocity process updating was enhanced by the method suggested in this work, and the most effective particle classification was used to determine the new particle position^163^. Using biomass characteristics and pyrolysis process factors, Muzammil Khanet al. (2022) have created a combined structure of metaheuristic algorithms and artificial neural networks (ANNs) for the forecasting of biochar yield^164^. Research on PV modelling is becoming increasingly popular. For PV systems to be designed and controlled effectively, PV models must be accurate. Thereafter, many static and dynamic models were created to simulate the current flow in solar cells. Considering these particulars, Shuhui Wang et al. (2021)^165^ suggest that the parameter extraction problem be solved using a hybrid Rao_1 Optimisation Algorithm (hROA).

Two parameter-free best-worst optimizers (BWOs) were proposed by Zohaib Hussain Leghari et al. (2022) to enhance the exploration and exploitation capabilities of the Rao-1 and Jaya algorithms^166^. Karpagalingam Thirumoorthy et al. (2022) proposed a hybrid feature selection (filter-wrapper) technique based on the Rao optimization method and multi-criteria decision-making (MCDM) method^167^ to improve the accuracy of software defect prediction. Yu-Jun Zhang et al. (2022) offer EHRJAYA, Rao-1, and a self-adaptive hybrid JAYA strategy for categorization learning. It can be used to handle challenging engineering optimization problems in the real world and large-scale numerical concerns^168^.

Radiation is a constant part of the workplace for those who work in nuclear power plants. To expose them to the least amount of radiation possible, it is crucial to plan the least dose course. However, it can be difficult to choose the approach with the least amount of dose in an environment with difficult obstacles or confined passageways. To resolve this issue, Kwon Ryong Hong et al. (2022) suggested a meta-heuristic approach. A new obstacle detour algorithm and Rao-combined artificial bee colony (RABC) algorithm are proposed^169^. Complex modern machining processes such as electric discharge machining (EDM) necessitate the application of sophisticated modeling and optimization methods. Initially, the experiment was designed, and the training data set was created using the response surface methodology (RSM). The same training data set was used to create and optimize the Rao artificial neural network (ANN) model. Rao has finally been given an ANFIS (adaptive neuro-fuzzy inference system) model^170^.

A hybrid approach that combines the SA and RA algorithms is proposed by Santos Kumar Baliarsingh et al. (2021)^171^ to select the optimal gene subset and classify cancer. A computerized approach based on the novel hybrid algorithm CSARao-1 is proposed by Walid Tadj et al. (2021). The Rao-1 and the Crow Search approach (CSA), two contemporary metaheuristic algorithms, are combined in this approach for the optimum estimation of aquifer parameters, which reaps the benefits of both techniques^172^. Sanjib Kumar Nayak et al. (2021) created a hybrid ANN employing the Rao algorithm (RA + ANN) to accurately predict six well-known cryptocurrencies: Ethereum, Litecoin, Bitcoin, CMC 200, Tether, and Ripple^173^. Likewise, several researchers employed hybrid Rao algorithms for solving various Science and Engineering optimization problems. These are k-NN-based Rao algorithms^174^. ANN-based Rao Algorithms^175^. An artificial neural network including a back propagation-based Rao algorithm^176^. Non-dominant sorting based on Rao algorithms^177^. Hybrid Jaya and Rao algorithms^178^, GPU Based Hybrid Best- Worst-Play (BWP) algorithm^179^.

**Table 6:** Applications of hybrid Rao algorithm.

Refs No.	Year	Method	Application	Contribution
^161^	2023	Discrete Rao-Combined ABC algorithm	Facility inspection	Problem of minimum dosage path planning achieved for facility inspection
^162^	2021	Rao-NM algorithm	Image template matching,	Iissue of picture template matching. solved
^163^	2021	Particle Swarm Optimization (PSO) with Rao algorithm	Classification of Wisconsin Breast Cancer Dataset	Wisconsin Breast Cancer Dataset to resolve the classification problem achieved
^164^	2022	ANN with Rao-2 algorithm	Prediction of biochar yield	Biochar yield. forecasted
^165^	2021	Hybrid Rao_1 Optimization Algorithm,	Static and dynamic solar photovoltaic models	Parameters of solar photovoltaic (PV) models that are both static and dynamic are calculated
^166^	2022	BWOs that combine the searching capabilities of Jaya and Rao-1 algorithms	Distribution Networks	The issue of power distribution networks’ concurrent distribution of shunt capacitors (SCs) and distributed generators (DGs) handled
^167^	2022	MCDM + RAO	Feature selection	Precision of software defect prediction by feature selection improved
^168^	2022	Self-adaptive classification learning hybrid JAYA and Rao-1 algorithm (SCLHJ-Rao)	Large-scale numerical and engineering problems	Significant engineering and numerical issues handled
^169^	20,222	Rao-combined ABC algorithm	Dose path planning in complex radioactive environment	Issue of arranging an inspection robot’s least dosage path in intricate radioactive surroundings solved
^170^	2021	Rao algorithm and Jaya algorithm	EDM	EDM surface roughness modelling and optimization achieved
^171^	2021	SARA (Self-adaptive Antlion Rao Algorithm), a memetic algorithm	High-dimensional biomedical data	Biomedical data with large dimensions handled
^172^	2021	CSA and Rao-1 algorithm	Estimating confined and leaky aquifers parameters	Transient time-drawdown data to estimate the hydraulic characteristics of leaky and constrained aquifers achieved
^173^	2022	Rao Algorithm-Based Artificial Neural Networks (ANNs)	Price prediction	Cryptocurrency closing values simulated and predicted
^174^	2024	modified Rao optimization method with a k-Nearest Neighbors (k-NN) classifier	Truss structures	Best discrete truss structure sizing calculated
^175^	2024	Artificial Neural Networks (ANNs) with Rao algorithms	Forecasting of Water Demand	Istanbul’s water consumption predicted
^176^	2024	ANN-Rao_1, ANN_Rao2 and ANN-Rao_3	Hydroelectric energy generation	The amount of energy generated by hydroelectric power calculated
^177^	2023	NDS-Rao-2 a	Construction scheduling	Multi-objective time-cost trade-off optimization issue resolved
^178^	2023	binMOJaya, binMORao1 and binMORao2	Project selection	Issue of multi-objective project selection handled
^179^	2023	Jaya and Rao-l	Nonlinear Equations	Large-scale nonlinear equation problems solved

## Proposed hybrid Rao algorithm (HRA) optimization algorithm

### Formulation of conventional Rao

A class of metaphor-free optimisation methods called the Rao algorithms was introduced in 2020 by Ravipudi Venkata Rao. The Rao Algorithms use accidental interactions among potential solutions to swiftly determine the optimal solution by combining the best and worst answers from each iteration. The algorithm uses two control parameters: population size and function evaluations, both easily adjustable^59^. Numerous characteristics, such as likelihood, intensity, cohesiveness, and other variables that are infamously hard to adjust, are eliminated from the earlier metaphor-based algorithms. Three variants of the Rao algorithms exist: Rao-1, Rao-2, and Rao-3. Each of these algorithms uses one of the three equations given below:

1 $$\:{X}_{j,\:k,\:i}^{{\prime\:}}=\:{X}_{j,k,i}+\:{r}_{1,\:j,i}\left({X}_{j,best,i}-\:{X}_{j,worst,i}\right)$$

2 $$\:{\:X}_{j,\:k,\:i}^{{\prime\:}}=\:{X}_{j,k,i}+\:{r}_{1,\:j,i}\left({X}_{j,best,i}-\:{X}_{j,worst,i}\right)\:+\:{r}_{2,j,i\:}(\left|{X}_{j,k,i}\:or\:{X}_{j,l,i}\right|-\left|{X}_{j,l,i}\:or\:{X}_{j,k,i}\right|)$$

3 $$\:{X}_{j,\:k,\:i}^{{\prime\:}}=\:{X}_{j,k,i}+\:{r}_{1,\:j,i}\left({X}_{j,best,i}-\:\left|{X}_{j,worst,i}\right|\right)+\:{r}_{2,j,i}\:\left(\left|{X}_{j,k,i}\:or\:{X}_{j,l,i}\right|-\left({X}_{j,l,i}\:or\:{X}_{j,k,i}\right)\right)$$

In this context, for the *i*^*th*^ iteration, the symbols $\:{X}_{j,best,i}$ and $\:{X}_{j,worst,i}$ denote the best candidate and the worst candidate, respectively, as the value of variable j. After the equation, the variable $\:{X}_{j,\:k,\:i}^{{\prime\:}}$ stands for the updated value, where both random variables $\:{r}_{1,\:j,i}\:$and $\:{r}_{2,j,i\:}$ are generated uniformly at random in the interval [0,1]. In the expression, $\:\left|{X}_{j,k,i}\:or\:{X}_{j,l,i}\right|$, the candidate solution *k* is contrasted with a different candidate i, which is chosen at random from the population of candidates. When * k* is a better fit than i, the term $\:\left|{X}_{j,k,i}\right|$ is chosen. If not, the $\:\left|{X}_{j,1,i}\right|$is picked. About the second term ($\:{X}_{j,1,i}\:or\:{X}_{j,k,i}$) the same rule is used^60^.

### Formulation of proposed Hybrid Rao algorithm (HRA)

**Fig. 4:**
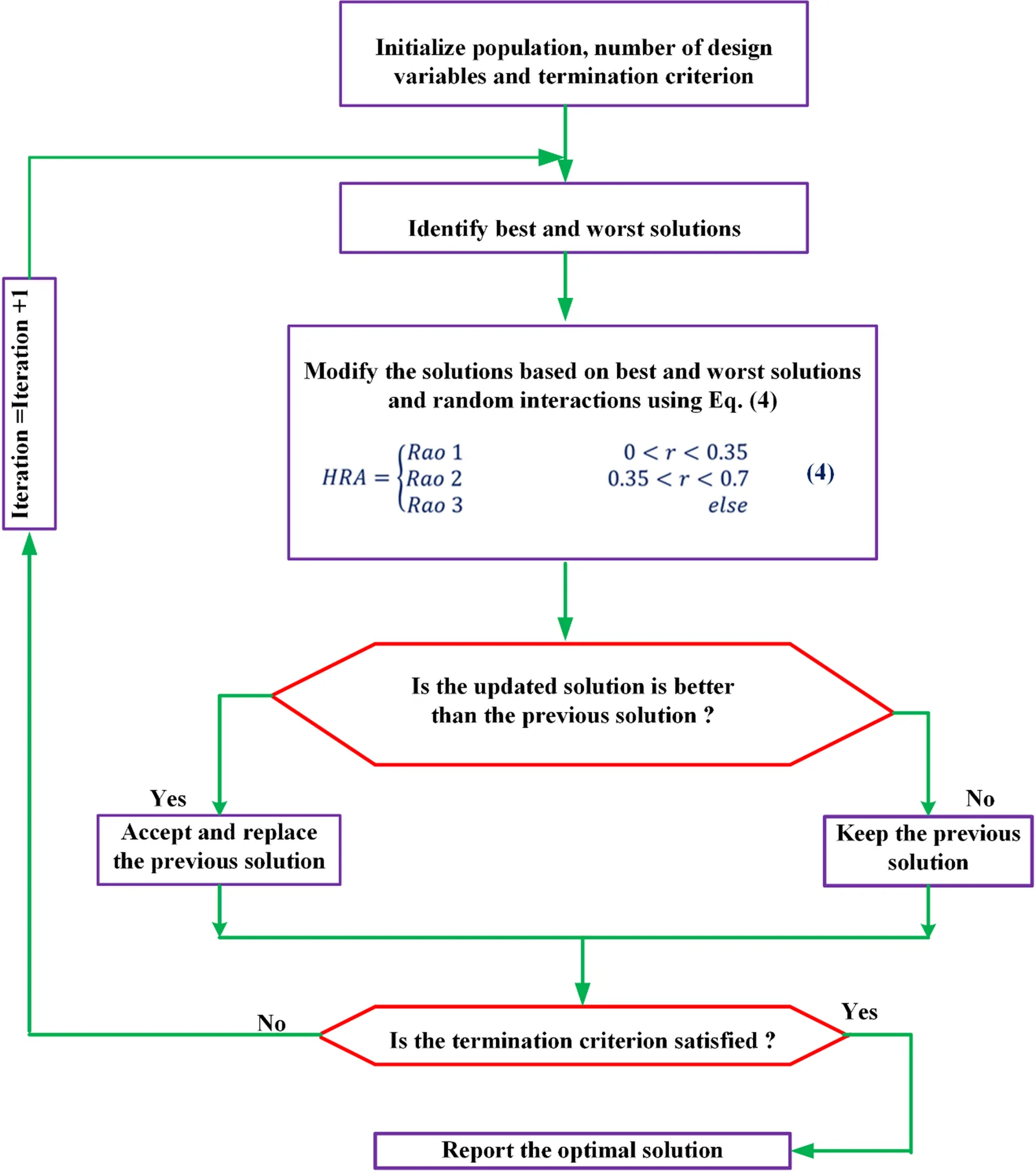
A flowchart of the proposed HRA algorithm.

Ravipudi Venkata Rao put up three straightforward optimization methods devoid of metaphors in 2020. To reach more favorable regions of the search space, the Rao algorithms can increase the gap with non-optimal answers and modify the search depending on inherited values drawn to the global best solution. As a result, the algorithms can search a large portion of the search space. In terms of achieving superior outcomes for resolving the mathematical benchmark problems, the three suggested algorithms can be considered competitive with the state-of-the-art optimization algorithms. However, it is observed that for complex real-world optimization problems, sometimes any single or all three variants of Rao algorithms are stuck in local optima. A flexible combination of the Rao algorithms (Rao-1, Rao-2, and Rao-3) may be able to overcome their common weaknesses by using their unique strengths. Therefore, this work offers a hybrid Rao algorithm (HRA) that prevents premature convergence and significantly boosts the efficiency of Rao algorithms, addressing the drawbacks of traditional Rao algorithms. Figure 4 presents a flowchart of the suggested HRA method.

The HRA employs a probabilistic switching mechanism among Rao-1, Rao-2, and Rao-3, the objective is not merely to combine existing algorithms, but to achieve a dynamic balance between exploration and exploitation by leveraging their complementary search behaviors. Specifically, Rao-1 is more exploration-oriented, Rao-2 provides a balanced search, and Rao-3 emphasizes exploitation. The probabilistic framework enables adaptive transitions among these behaviors during the optimization process, which contributes to the observed performance improvements. The probability thresholds (0.35 and 0.7) were determined through extensive empirical experimentation. During the experimentation phase, multiple trials were conducted using different combinations of threshold values to evaluate their impact on the performance of the proposed HRA. It was observed that the selected values (0.35 and 0.7) consistently yielded better and more stable results across the considered benchmark problems. The solution update equation for the combined Rao 1, Rao 2, and Rao 3 algorithms can be utilized to generate the hybrid Rao algorithms. The following is the suggested structure for the proposed HRA algorithm:

4 $$\:HRA=\left\{\begin{array}{c}Rao\:1\:\:\:\:\:\:\:\:\:\:\:\:\:\:\:\:\:\:\:\:\:\:\:\:\:\:\:\:\:\:\:\:0<r<0.35\\\:Rao\:2\:\:\:\:\:\:\:\:\:\:\:\:\:\:\:\:\:\:\:\:\:\:\:\:\:\:\:\:\:0.35<r<0.7\\\:Rao\:3\:\:\:\:\:\:\:\:\:\:\:\:\:\:\:\:\:\:\:\:\:\:\:\:\:\:\:\:\:\:\:\:\:\:\:\:\:\:\:\:\:\:\:\:\:\:\:\:\:else\end{array}\:\right.$$

**Figure:**
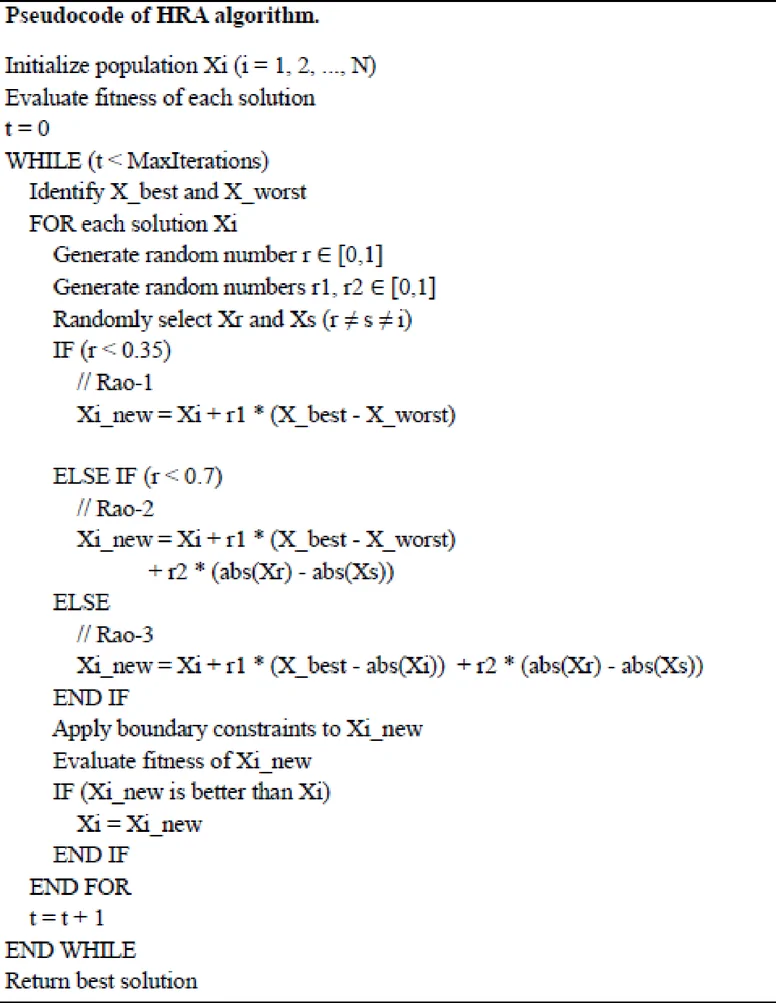

### Problem formulation

The ORPD problem is expressed mathematically as follows^180^:

5 $$\:Min\:R\:(a,\:b)$$

Subject to;

6 $$\:G\:(a,\:b)\hspace{0.17em}=\hspace{0.17em}0$$

and

7 $$\:H\:(a,\:b)\hspace{0.17em}\le\:\hspace{0.17em}0$$

where *R (a*,* b*) indicates the objective function that must be minimised. The set of dependent variables is represented by ‘a’ and ‘b’. In this case, *a* can be written as

8 $$\:{\mathrm{a}}^{\mathrm{T}}=[{{P}_{{g}_{1}},\mathrm{V}}_{1}\dots\:{\mathrm{V}}_{\mathrm{N}\mathrm{L}\mathrm{B}},{\mathrm{Q}}_{\mathrm{g}1},\dots\:{\mathrm{Q}}_{\mathrm{g}\mathrm{N}\mathrm{G}},{S}_{1},\dots\:{S}_{\mathrm{n}\mathrm{t}\mathrm{l}}]$$

while, *b* can be expressed as

9 $$\:{\mathrm{b}}^{\mathrm{T}}=[{\mathrm{V}}_{{\mathrm{g}}_{1}}\dots\:{\mathrm{V}}_{{\mathrm{g}}_{\mathrm{N}\mathrm{G}\mathrm{N}}},{\mathrm{Q}}_{{\mathrm{C}}_{1}}\dots\:{\mathrm{Q}}_{{\mathrm{C}}_{\mathrm{N}\mathrm{C}}},{\mathrm{T}}_{1}\dots\:{\mathrm{T}}_{\mathrm{N}\mathrm{T}\mathrm{R}}]$$

- **Constraints**:

- **Equality Constraints (G)**.

The power flow equations will define the equality restrictions. Reactive and Active power flow equations can be distinguished. The following are the equations for the flow of reactive and active power.

10 $$\:{0\:\:=\:\:\:\:\:\:\:\mathrm{P}}_{\mathrm{g}\mathrm{i}}-{\mathrm{P}}_{\mathrm{d}\mathrm{i}}-{\mathrm{P}}_{\mathrm{L}\mathrm{o}\mathrm{s}\mathrm{s}}$$

11 $$\:{0\:\:=\:\:\:\:\:\:\mathrm{Q}}_{\mathrm{g}\mathrm{i}}-{\mathrm{Q}}_{\mathrm{d}\mathrm{i}}-\:\:\:{\mathrm{Q}}_{\mathrm{L}\mathrm{o}\mathrm{s}\mathrm{s}}$$

b) **Inequality Constraints (H)**:

Power systems are made up of numerous components and equipment. There are limitations to how these devices can operate. These devices’ minimum and maximum limits are known as inequality constraints.

- *Security Constraints*:

12 $$\:{\mathrm{V}}_{{\mathrm{L}}_{\mathrm{k}}}^{\mathrm{m}\mathrm{i}\mathrm{n}}\le\:{\mathrm{V}}_{{\mathrm{L}}_{\mathrm{k}}}\le\:{\mathrm{V}}_{{\mathrm{L}}_{\mathrm{k}}}^{\mathrm{m}\mathrm{a}\mathrm{x}}\:\:\:\:\:\:\:\:\mathrm{k}=1\dots\:\dots\:\mathrm{N}\mathrm{L}\mathrm{B}$$

13 $$\:{\mathrm{S}}_{{\mathrm{l}}_{\mathrm{k}}}\le\:{\mathrm{S}}_{{\mathrm{l}}_{\mathrm{k}}}^{\mathrm{m}\mathrm{a}\mathrm{x}}\:\:\:\:\:\:\:\:\:\:\:\mathrm{k}=1\dots\:\dots\:\mathrm{n}\mathrm{t}\mathrm{l}$$

- *Transformer Constraints*:

14 $$\:{\mathrm{T}}_{\mathrm{k}}^{\mathrm{m}\mathrm{i}\mathrm{n}}\:\le\:{\mathrm{T}}_{\mathrm{k}}\le\:{\mathrm{T}}_{\mathrm{k}}^{\mathrm{m}\mathrm{a}\mathrm{x}}\:\:\:\mathrm{k}=1\dots\:\dots\:\mathrm{N}\mathrm{T}\mathrm{R}$$

- *Shunt VAR compensator constraints*:

15 $$\:{\mathrm{Q}}_{{\mathrm{C}}_{\mathrm{k}}}^{\mathrm{m}\mathrm{i}\mathrm{n}}\le\:{\mathrm{Q}}_{{\mathrm{C}}_{\mathrm{k}}}\le\:{\mathrm{Q}}_{{\mathrm{C}}_{\mathrm{k}}}^{\mathrm{m}\mathrm{a}\mathrm{x}}\:\:\:\mathrm{k}=1\dots\:\dots\:\mathrm{N}\mathrm{G}\mathrm{N}$$

- *Generators constraints*:

16 $$\:{\mathrm{V}}_{{\mathrm{g}}_{\mathrm{k}}}^{\mathrm{m}\mathrm{i}\mathrm{n}}\le\:{\mathrm{V}}_{{\mathrm{g}}_{\mathrm{k}}}\le\:{\mathrm{V}}_{{\mathrm{g}}_{\mathrm{k}}}^{\mathrm{m}\mathrm{a}\mathrm{x}}\:\:\:\:\:\:\:\:\mathrm{k}=1\dots\:...\mathrm{N}\mathrm{G}\mathrm{N}$$

17 $$\:{\mathrm{Q}}_{{\mathrm{g}}_{\mathrm{k}}}^{\mathrm{m}\mathrm{i}\mathrm{n}}\le\:\:{\mathrm{Q}}_{{\mathrm{g}}_{\mathrm{k}}}\le\:{\mathrm{Q}}_{{\mathrm{g}}_{\mathrm{k}}}^{\mathrm{m}\mathrm{a}\mathrm{x}}\:\:\:\:\:\mathrm{k}=1\dots\:\dots\:\mathrm{N}\mathrm{G}\mathrm{N}$$

Where P_gi_ and Q_gi_ denote the generator’s real and reactive power output of the *i*^*th*^ bus, P_di_ and Q_di_ denote the bus real and reactive power demands at the *i*^*th*^ bus, $\:{\mathrm{Q}}_{{\mathrm{C}}_{\mathrm{k}}}$ denotes the limits on the output from shunt VAR compensators at the *k*^*th*^ bus, V_Lk_ denotes the limits on the load bus voltages at the *k*^*th*^ bus. $\:{\mathrm{S}}_{{\mathrm{l}}_{\mathrm{k}}}$ denotes an MVA limit on the line flow, NTR is the number of regulating transformers, and NGN and NLB denote the count of generator and load buses, respectively.

### Incorporation of constraints

To ensure feasible solutions, inequality constraints are incorporated into the fitness function (18):

18 $$\:{\:\mathrm{R}}_{\mathrm{a}\mathrm{u}\mathrm{g}}=\mathrm{R}(\mathrm{a},\mathrm{b})+{\mathrm{P}}_{1}.\mathrm{H}\left({\mathrm{P}}_{{\mathrm{G}}_{1}}\right)+{\mathrm{P}}_{2}{\sum\:}_{\mathrm{i}=1}^{\mathrm{N}\mathrm{G}\mathrm{N}}\mathrm{H}\left(\:{\mathrm{Q}}_{{\mathrm{G}}_{\mathrm{i}}}\right)+{\mathrm{P}}_{3}{\sum\:}_{\mathrm{i}=1}^{\mathrm{N}\mathrm{L}\mathrm{B}}\mathrm{H}\left(\:{\mathrm{V}}_{{\mathrm{L}}_{\mathrm{i}}}\right)+{\mathrm{P}}_{4}{\sum\:}_{\mathrm{i}=1}^{\mathrm{n}\mathrm{t}\mathrm{l}}\mathrm{H}\left({\:\mathrm{S}}_{{\mathrm{l}}_{\mathrm{i}}}\right)$$

Where$\:{\:\mathrm{P}}_{1}$,$\:{\mathrm{P}}_{2}$, $\:{\mathrm{P}}_{3}$ and $\:{\mathrm{P}}_{4}$ represent penalty factors for limit violations which are chosen by the user.

### Objective function formulation

#### • Case 1

• **Voltage profile improvement (VPI)**.

This goal’s main objective is to reduce each load bus’s voltage variance from 1.0 p.u. The mathematically formulation of given function as fellow^181^:

19 $$\:{\mathrm{F}}_{\mathrm{T}\mathrm{V}\mathrm{D}}\left(\mathrm{a},\mathrm{b}\right)={\sum\:}_{\mathrm{i}\epsilon\mathrm{N}\mathrm{L}\mathrm{B}}^{\:\:}\left|{\mathrm{V}}_{\mathrm{i}}-1\right|$$

#### • Case 2

• **Real power loss minimization (RPLM)**.

As a prime goal function, RPLM was chosen in Case 2^182^. F_Loss_ can be stated mathematically as Eq. (20).

20 $$\:{F}_{LOSS}\left(a,b\right)={\sum\:}_{i=1}^{NB}{P}_{gi}-{\sum\:}_{i=1}^{NB}{P}_{di}$$

## Outcomes of the simulation and discussions

This section presents a comparative performance analysis of the proposed HRA method against five widely used benchmark functions and classical Rao algorithm variants. To evaluate its effectiveness, the HRA algorithm is compared with Rao-1, Rao-2, and Rao-3 algorithms under identical conditions, where all methods are executed with a population size of 30 for 100 iterations. The details of the benchmark functions are provided in Appendix A. The comparative results, summarized in Table 7, demonstrate that the proposed HRA method outperforms all three classical Rao variants across the considered benchmark functions. The HRA algorithm was implemented in MATLAB 13a on a personal computer equipped with a 64-bit operating system, 8 GB RAM, and an Intel Core i7 processor.

**Table 7:** HRA algorithm results for benchmark functions that were taken into consideration (30000 function assessments).

Function	F_Min_		Rao-1^59^	Rao-2^59^	Rao-3^59^	Proposed HRA
$\:{\mathbf{F}}_{1}\left(\mathbf{x}\right)$	0	W	3.28E-21	3.47E-11	6.29E-41	4.32E-58
		B	4.84E-25	1.40E-15	1.58E-50	8.91E-72
		M	3.59E-22	3.57E-12	6.71E-42	5.69E-54
		SD	7.33E-22	7.95E-12	1.56E-41	2.04E-69
		MFE	29,998	29,953	29,991	30,000
$\:{\mathbf{F}}_{2}\left(\mathbf{x}\right)$	0	W	7.60E-11	10.0012	2.10E-19	2.10E-38
		B	2.04E-15	0.00012	6.32E-24	6.32E-30
		M	4.07E-12	0.67817	9.33E-21	9.33E-33
		SD	1.40E-11	2.53445	3.84E-20	3.84E-26
		MFE	29,994	29,882	29,983	29,995
$\:{\mathbf{F}}_{3}\left(\mathbf{x}\right)$	−12,569	W	−3879.4985	−5960.01496	−5751.10732	−5928.41209
		B	−10250.8258	−12352.3469	−12135.20714	−12315.19834
		M	−8685.17016	−8757.58136	−9664.70182	−9811.20451
		SD	1690.54881	1896.34347	1544.65568	1611.32214
		MFE	21,166	22,377	28,385	27,943
$\:{\mathbf{F}}_{4}\left(\mathbf{x}\right)$	0	W	183.60571	232.79199	197.125802	85.357635
		B	25.86892	68.121702	29.889988	18.554324
		M	87.01355	148.94949	84.122877	65.189547
		SD	32.31749	41.52665	38.179200	29.976410
		MFE	26,015	24,754	27,934	26,613
$\:{\mathbf{F}}_{5}\left(\mathbf{x}\right)$	0	W	2.131898	1.350810	3.24E-07	5.98E-14
		B	4.41E-07	1.43E-02	7.57E-10	7.12E-19
		M	0.619739	0.170688	7.97E-08	3.54E-16
		SD	0.695792	0.318320	8.69E-08	9.02E-14
		MFE	29,929	29,881	29,919	29,988

For the OPRD problem, several case studies are conducted in this paper, as summarized in Table 8.

**Table 8:** Various case studies of OPF problem investigated in this work.

IEEE 30-bus system	
Case 1	Minimization of Total Voltage Deviation
Case 2	Real Power Loss Minimization
**IEEE 57-bus system**	
Case 3	Minimization of Total Voltage Deviation
Case 4	Real Power Loss Minimization

### ORPD (Optimal reactive power dispatch)

#### Test system 1: IEEE 30-bus system

This study solves the ORPD problem for the IEEE 30-bus system using the proposed HRA approach. The IEEE 30 bus system specifications are available in^180^. The maximum number of iterations *(It_max)* = 150 and population size *(NP)* = 30 for the ORPD problem have yielded the best results, even though many trials have been conducted; these results are presented in this article. To solve the ORPD problem, two target functions—actual voltage profile enhancement and power loss minimization are considered in this work.

#### Case study 1: minimization of total voltage deviation

**Fig. 5:**
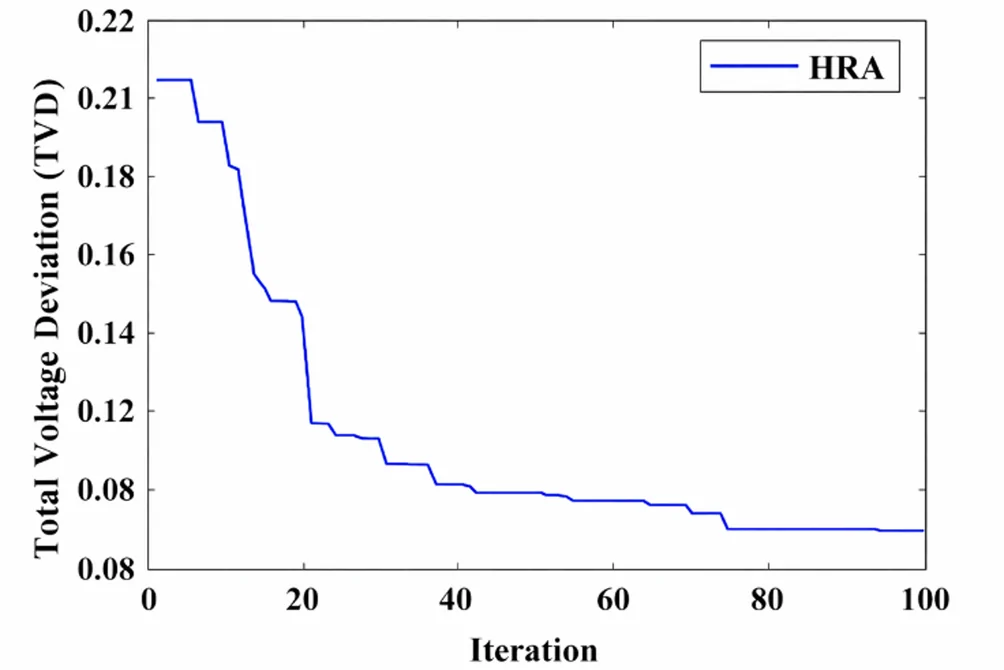
Convergence of case 1.

Table 9 displays the optimal control variable values, and Fig. 5 displays the convergence characteristics that the HRA method in Case 1 produced. Table 10 presents a comparison between the Case 1 simulation results produced using the suggested HRA, Rao-1, Rao-2, and Rao-3 algorithms, and other known methods that have been studied in recent works. The efficiency of the HRA algorithm in comparison to the numerical findings illustrate the effectiveness of Rao-1, Rao-2, and Rao-3 algorithms along with other reported approaches for this case. The HRA algorithm provides the least voltage deviation in comparison to the other Rao variations’ methods.

**Table 9:** The optimum values of control variables of Case 1 & Case 2.

S. No	Control Variable	Initial Case	Case 1	Case 2
PV bus Voltage				
**1**	$\:{\boldsymbol{V}}_{1}$	1.05	0.99776	1.07129
**2**	$\:{\boldsymbol{V}}_{2}$	1.04	1.00407	1.02183
**3**	$\:{\boldsymbol{V}}_{5}$	1.01	1.01951	1.03988
**4**	$\:{\boldsymbol{V}}_{8}$	1.01	1.04080	1.03978
**5**	$\:{\boldsymbol{V}}_{11}$	1.05	1.01510	1.09471
**6**	$\:{\boldsymbol{V}}_{13}$	1.05	1.02903	1.05772
**Transformer tap settings**				
**7**	$\:{\boldsymbol{T}}_{11}$	1.078	1.03031	1.03456
**8**	$\:{\boldsymbol{T}}_{12}$	1.069	0.90000	0.95159
**9**	$\:{\boldsymbol{T}}_{15}$	1.032	1.02219	0.99360
**10**	$\:{\boldsymbol{T}}_{36}$	1.068	0.95995	0.97825
***VAR setting***				
**11**	$\:{\boldsymbol{Q}\boldsymbol{s}\boldsymbol{h}}_{10}$	0.0	0.05000	0.02316
**12**	$\:{\boldsymbol{Q}\boldsymbol{s}\boldsymbol{h}}_{12}$	0.0	0.03747	0.02506
**13**	$\:{\boldsymbol{Q}\boldsymbol{s}\boldsymbol{h}}_{15}$	0.0	0.04924	0.04567
**14**	$\:{\boldsymbol{Q}\boldsymbol{s}\boldsymbol{h}}_{17}$	0.0	0.00000	0.04971
**15**	$\:{\boldsymbol{Q}\boldsymbol{s}\boldsymbol{h}}_{20}$	0.0	0.04998	0.04369
**16**	$\:{\boldsymbol{Q}\boldsymbol{s}\boldsymbol{h}}_{21}$	0.0	0.05000	0.04960
**17**	$\:{\boldsymbol{Q}\boldsymbol{s}\boldsymbol{h}}_{23}$	0.0	0.04959	0.02465
**18**	$\:{\boldsymbol{Q}\boldsymbol{s}\boldsymbol{h}}_{24}$	0.0	0.0IEq414929	0.05000
**19**	$\:{\boldsymbol{Q}\boldsymbol{s}\boldsymbol{h}}_{29}$	0.0	0.02022	0.02817
**Total Voltage Deviation (p.u)**		1.1601	**0.09003**	0.8639
**Real Power Loss** **(MW)**		5.8423	6.20423	**4.87437**

**Table 10:** Results comparison for Case 1.

S. No	Algorithms	Voltage (pu)	CT
1	**HRA**	**0.09003**	**83.6541**
2	Rao-1	0.102	84.2567
3	Rao-2	0.0960	85.0156
4	Rao-3	0.0961	83.7857
5	GSA^183^	0.118	-
6	BFO^183^	0.149	-
7	DE^183^	0.09283	-
8	HFA^183^	0.098	-
9	SaDE^183^	0.09682	-
10	ABC^183^	0.135	-
11	PSO^183^	0.1535	-
12	ARO^184^	0.1228	-
13	GBO^184^	0.1226	-
14	AROGBO^184^	0.1224	-
15	RSO^185^	0.2936	-
16	PDO^185^	0.1410	-
17	ROA^185^	0.1267	-
18	SPO^185^	0.1247	-
19	MWAA^186^	0.1240	-
20	WAA^186^	0.3701	-

CT=Computation time in second.

#### Case study 2: real power loss minimization (RPLM)

**Fig. 6:**
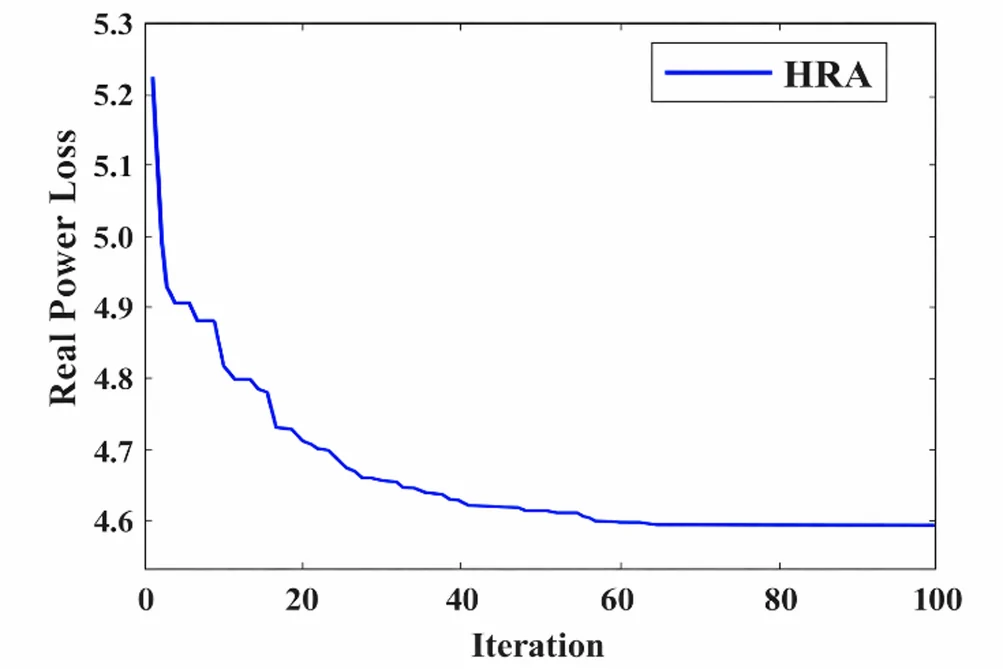
Convergence of case 2.

While the Rao-1, Rao-2, and Rao-3 methods achieved minimum power losses of 3.1086 MW, 3.3041 MW, and 3.3041 MW, respectively, the HRA algorithm achieved the lowest power loss of 3.0675 MW. Table 11 juxtaposes the Case 2 simulation outcomes derived from the HRA, Rao-1, Rao-2, and Rao-3 methods with additional documented techniques enumerated in recent publications. Table 11 displays the HRA algorithm findings and the best control variable values. When compared to the other methods, numerical data unequivocally demonstrate that the HRA algorithm offers the least amount of power loss in this situation. Figure 6 depicts the power loss characteristic of Case 2. Table 9 summarize the optimal configurations of the control variables for this case.

**Table 11:** Results comparison for Case 2.

S. No	Algorithms	Real Power loss (MW)	CT
1	**HRA**	**4.87437**	**82.9874**
2	Rao 1	4.87788	**83.9712**
3	Rao 2	4.8736	**82.9964**
4	Rao 3	4.87566	**83.6789**
5	SaDE^183^	4.8928	-
6	DE^183^	4.8750	-
7	ARO^184^	4.9451	-
8	GBO^184^	4.9452	-
9	AROGBO^184^	4.9450	-
10	RSO^185^	5.133	-
11	PSOGWO^187^	5.090	-

CT=Computation time in second.

### Test system 2: IEEE 57-bus system

The effectiveness of the HRA algorithm was evaluated on the well-known IEEE 57 bus system, a benchmark for power system optimization. Detailed system parameters and operating limits of the control variables were adopted from standard benchmark data reported in^188^.

#### Case study 3

**Fig. 7:**
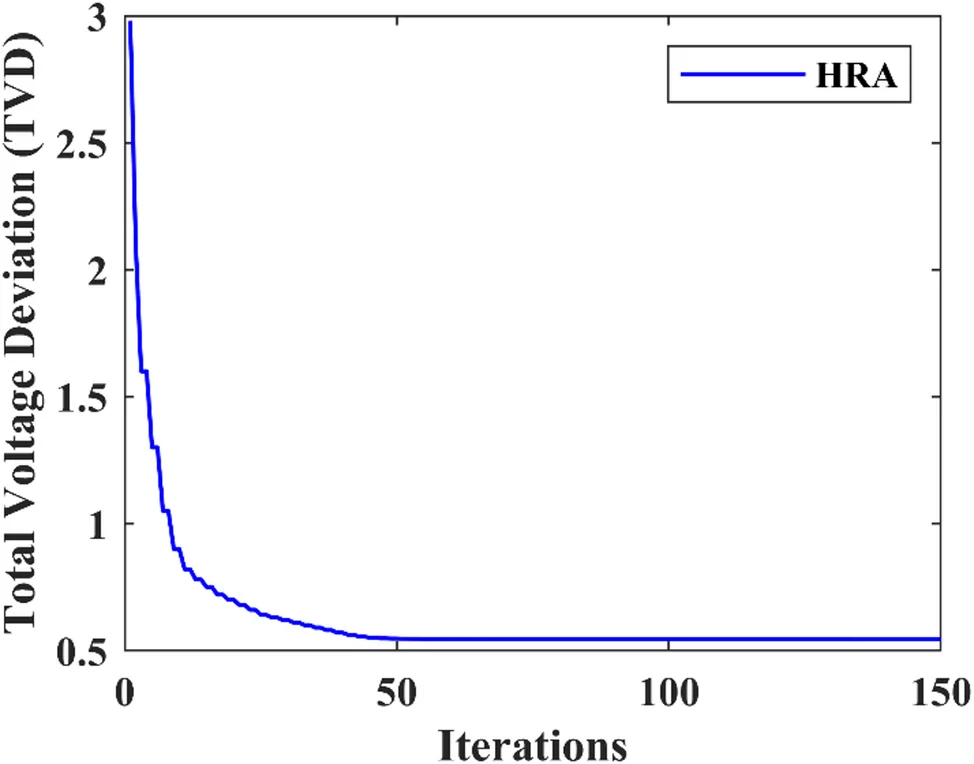
Convergence of case 3.

In this case, the proposed HRA has been applied to solve ORPD problem considering the objective function consisting of the total voltage deviation. The minimum voltage deviation obtained by the proposed HRA algorithm are 0.5472 p.u. The numerical results of proposed algorithm along with optimal control variable settings are shown in Table 12. In this case, the voltage profile is significantly improved in comparison of initial case. In initial case, the total voltage deviation is 1.2236 p.u, which is reduced to 0.5472 p.u. in case 3. The numerical results obtained by HRA algorithm and other reported results available in recent literature are compared in Table 13. It can be observed from Table 13 that the HRA algorithm provides the minimum value of the objective function. This reveals the effectiveness of the HRA algorithm compared with other competitors. Convergence characteristics of proposed HRA algorithms is shown in Figure. 7.

**Table 12:** Optimum values of control variables of CASE 3- Case 4 OF IEEE 57-bus system.

S. No	Control variable	Initial Case	Case-4	Case-5
PV bus Voltage				
**1**	**V**_**G1**_	1.04	1.0185	1.067
**2**	**V**_**G2**_	1.01	0.9208	1.0708
**3**	**V**_**G3**_	0.985	1.0092	1.058
**4**	**V**_**G6**_	0.98	1.0017	1.0601
**5**	**V**_**G8**_	1.005	1.0087	1.0717
**6**	**V**_**G9**_	0.98	1.0514	1.0551
**7**	**V**_**G12**_	1.015	1.0068	1.0509
Transformer tap settings				
**8**	**T**_**4−18**_	0.97	0.9493	0.9629
**9**	**T**_**4−18**_	0.978	1.0092	0.9738
**10**	**T**_**21−20**_	1.043	0.9709	1.0077
**11**	**T**_**24−25**_	1	1.075	1.085
**12**	**T**_**24−25**_	1	1.0781	1.0999
**13**	**T**_**24−26**_	1.043	1.0036	1.0311
**14**	**T**_**7−29**_	0.967	0.9996	0.998
**15**	**T**_**34−32**_	0.975	0.9198	0.9526
**16**	**T**_**11−41**_	0.955	0.9001	0.9
**17**	**T**_**15−45**_	0.955	0.9828	0.9853
**18**	**T**_**14−46**_	0.9	0.9425	0.967
**19**	**T**_**10−51**_	0.93	1.008	0.9784
**20**	**T**_**13−49**_	0.895	0.904	0.9387
**21**	**T**_**11−43**_	0.958	0.9739	0.9907
**22**	**T**_**40−56**_	0.958	1.0476	1.0003
**23**	**T**_**39−57**_	0.98	0.9012	0.9647
**24**	**T**_**9−55**_	0.94	1.0531	1.0025
***VAR setting***				
**25**	**Q**_**sh18**_	0.1	0.011	0.0029
**26**	**Q**_**sh25**_	0.059	0.2215	0.2697
**27**	**Q**_**sh53**_	0.063	0.2988	0.136
**Total Voltage Deviation (p.u)**		1.2236	**0.5472**	1.4758
**Real Power Loss** **(MW)**		27.8282	43.8278	**23.2195**

**Table 13:** Results comparison for Case 3.

S. No	Algorithms	Voltage (pu)	CT
1	**HRA**	**0.5472**	**98.6785**
2	Rao-1	0.5967	99.8745
3	Rao-2	0.5887	98.7987
4	Rao-3	0.5809	99.6089
5	ARO^184^	0.6255	-
6	GBO^184^	0.6255	-
7	AROGBO^184^	0.6143	-
8	ASNS^188^	0.6405	-
9	SNS^188^	0.6520	-
10	GB-WCA^189^	0.6501	-
11	WCA^189^	0.6631	-
12	CBA-IV^190^	0.6399	-
13	BA^190^	0.6331	-

CT=Computation time in second.

#### Case study 4

**Fig. 8:**
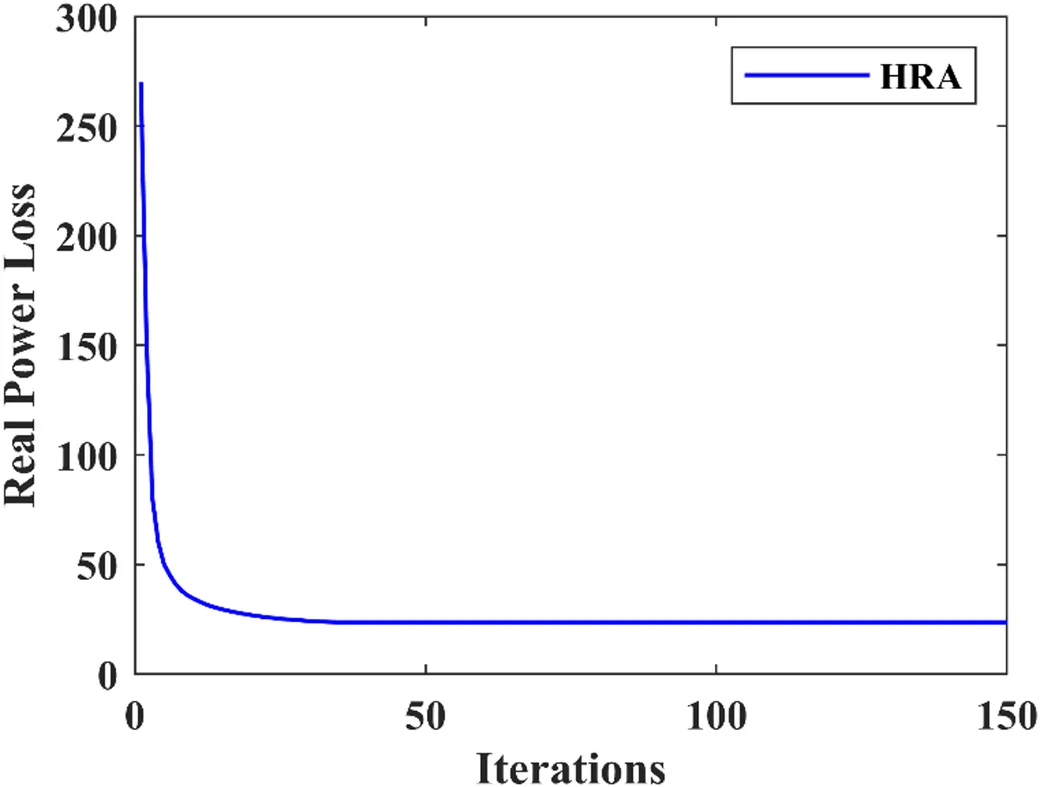
Convergence of case 4.

In this case the proposed HRA algorithm was used to solve the OPRD problem considering objective function that is, minimization real power loss. The simulation result obtained of real power loss by proposed HRA algorithm is 23.2195 MW. Figure 8 represent the convergence characteristic of HRA algorithm.Table 12 shows the control variables setting of this case. In comparison with other reported algorithms, proposed HRA algorithm offered the minimum value of objective function in case 4 and given in Table 14.

**Table 14:** Results comparison for Case 4.

S. No	Algorithms	Real power loss (MW)	CT
1	**HRA**	**23.2195**	**99.0989**
2	Rao 1	23.7098	99.8789
3	Rao 2	23.3909	100.0836
4	Rao 3	23.4276	100.9823
5	ARO^184^	23.6549	-
6	GBO^184^	23.6390	-
7	AROGBO^184^	23.6126	-
8	PDO^185^	50.5714	-
9	RSO^185^	32.6055	-
10	AVOA^185^	27.5168	-
11	ROA^185^	47.2455	-
12	SPO^185^	39.2672	-
13	ABC^191^	24.102	-
14	CGA^191^	25.744	-
15	HAS^191^	24.501	-
16	CLPSO^191^	24.515	-

CT=Computation time in second.

### Statistical analysis

The Friedman aligned rank test was applied to evaluate the performance of the HRA algorithms over 30 independent runs. The obtained average ranks are given in Table 15. The results clearly indicate that the HRA outperforms the other algorithms, achieving the best rank consistently across all runs.

**Table 15:** Average ranking of algorithms by friedman aligned test results.

Algorithm	Average rank
HRA	31.5
Rao-2	52.7
Rao-3	68.2
Rao-1	101.0

## Discussion

Complex optimization problems that involve multiple variables and objectives can be proficiently solved by using Rao algorithms. Because Rao algorithms can handle continuous, non-linear, and discrete objective functions with or without constraints, they have been effectively used to solve difficulties in a variety of scientific and technical domains.

The Rao algorithms occasionally experience premature convergence for complex optimization problems, just like other population-based algorithms. Modifying the original Rao algorithms’ structure is required to increase their capacity to address the problems. Four methods can be used to increase the effectiveness of Rao algorithms:

(i) by incorporating them with other AI paradigms e.g. (machine learning and Deep learning methods)
(ii) by hybridizing them with other Swarm Intelligence/nature-inspired optimization techniques.
(iii) by adding stochastic operators to balance the exploitation and exploration ability of the technique.
(iv) by strengthening the traditional Rao algorithms’ structure.

Rao algorithms are combined with other AI paradigms to boost their effectiveness. The hybridization improved the quality of the results without significantly adding complexity. These paradigms include fuzzy controllers, neural networks, feature selection algorithms, pattern recognition, etc. Rao algorithms and their variants are so easy to use that integrating them with other, far more sophisticated systems is the preferred approach. Given that the Rao algorithms yield some promising results across several research disciplines, further avenues for future improvements could be proposed.

The hybridization can also be used in combination with other global search techniques (other Swarm Intelligence/nature-inspired optimization) that don’t rely on Rao algorithms or specific local search techniques. It has been demonstrated that hybridization will improve Rao algorithms’ global search capacity. Heuristics are added to some variations of the algorithm, while modest adjustments are made to the program’s stages in other modifications. All of these were necessary to satisfy the requirements of the optimization problems. The efficiency of the standard Rao methods was greatly enhanced by incorporating the modifications to employ the other stochastic components, which allowed it to address the complex constraint multi-objective optimization problems. These modifications could be applied at the operator, population, or parameter levels. Rao algorithms’ general structure stayed the same, but many processes saw notable improvements in terms of reward and efficiency.

By integrating the advantages of each of the Rao algorithms—Rao-1, Rao-2, and Rao-3—this work has modified them and may be able to overcome their common shortcomings. The proposed hybrid Rao method aims to prevent sub-optimal outcomes by maintaining a variety of solutions during the search process and identifying near-global optimum solutions. The effectiveness of the recommended hybrid approach has been tested on both well-known mathematics problems and well-known power system optimization challenges. Five different benchmark (unimodal and multimodal) functions have been used to illustrate the efficacy of the proposed hybrid Rao algorithm. The numbers are shown in Table 13. The hybrid Rao approach outperforms all three of the recently released Rao algorithm variations, as demonstrated by the numerical findings in Table 13.

The suggested hybrid Rao algorithm solution produces statistically significant results in mathematical benchmark problems, as demonstrated by the lowest values of the hybrid Rao algorithm’s standard deviation, average, worst, and best. This confirms to the hybrid Rao algorithm’s ability to successfully tackle the complex constrained real-world optimization problem. Additionally, this work proposes a hybrid Rao algorithm to solve the RPD problem. The hybrid Rao technique that has been suggested is faster and less prone to local optima entrapment than the classic Rao algorithms, as demonstrated by the numerical results.

## Conclusions

The Rao algorithms are well-known parameter-less commanding global optimization approaches. Complex optimization issues involving multiple variables and objectives can be proficiently solved by using Rao algorithms. Although Rao algorithms have been successfully applied in numerous articles, there hasn’t been much focus on providing a complete summary of Rao algorithms. The purpose of this study is to provide current information on Rao algorithms that will be useful to researchers in this field. Like other population-based algorithms, the Rao algorithms may occasionally experience premature convergence. Therefore, the authors have developed a hybrid Rao algorithm in this work to get over the drawbacks of the traditional Rao algorithms. Using the merits of each Rao algorithm separately, a versatile combination of Rao-1, Rao-2, and Rao-3 may be able to overcome their common drawbacks. By finding near-global optimum solutions and preserving a range of solutions during the search process, the HRA seeks to avoid sub-optimal results. Numerical outcomes comparing the HRA to the classical Rao algorithms given in the recent literature show that the HRA provides better results than Rao algorithms for benchmark functions. Furthermore, by solving the RPD problem in the IEEE 30-bus and IEEE 57 bus systems, the proposed approach greatly reduced real power loss and improved the voltage profile. When compared to the optimization outcomes of the RPD problem, the hybrid Rao algorithm is more effective than the traditional (Rao-1, Rao-2, and Rao-3) approach and other reported methods.

The above-described study can be used to determine the following future directions:

- It might be feasible to conduct more research in the future on the application of HRA in domains of machine learning and computer vision.
- FACTS devices can be designed with dynamic models and placed in the best location to study how they affect the voltage stability of power systems and voltage profiles under both normal and extreme loading scenarios.
- Another avenue for further research is the incorporation of discrete control variables in the design of RPD problems, such as shunt compensators and transformer tap settings.

## Appendix A: Description of benchmark functions

The benchmark functions used in this study are standard mathematical optimization test functions commonly employed to evaluate the performance of metaheuristic algorithms. Five benchmark functions (F₁–F₅) are considered, which include both unimodal and multimodal functions.

- **Unimodal Functions**:

**F₁ (Sphere Function)**:

$$\:{F}_{1}\left(x\right)=\sum\:_{i=1}^{n}{x}_{i}^{2}$$

This function has a single global optimum and is used to evaluate the exploitation ability and convergence speed of the algorithm.

**F₂ (Sum and Product Function)**:

$$\:{F}_{2}\left(x\right)=\sum\:_{i=1}^{n}\mid\:{x}_{i}\mid\:+\prod\:_{i=1}^{n}\mid\:{x}_{i}\mid\:$$

This function tests the algorithm’s capability to handle simple but non-linear search landscapes.

- **Multimodal Functions**:

**F₃ (Schwefel Function)**:

$$\:{F}_{3}\left(x\right)=\sum\:_{i=1}^{n}-{x}_{i}\mathrm{s}\mathrm{i}\mathrm{n}\left(\sqrt{\mid\:{x}_{i}\mid\:}\right)$$

This function contains many local minima and is used to test exploration ability.

**F₄ (Rastrigin Function)**:

$$\:{F}_{4}\left(x\right)=\sum\:_{i=1}^{n}\left[{x}_{i}^{2}-10\mathrm{c}\mathrm{o}\mathrm{s}\left(2\pi\:{x}_{i}\right)+10\right]$$

This is a highly multimodal function used to evaluate the algorithm’s ability to avoid local optima.

**F₅ (Ackley Function)**:

$$\:{\mathrm{F}}_{5}\left(\mathrm{x}\right)=-20\mathrm{exp}\left(-0.2\sqrt{\frac{1}{\mathrm{n}}\sum\:_{\mathrm{i}=1}^{\mathrm{n}}{\mathrm{x}}_{\mathrm{i}}^{2}}\right)-\mathrm{exp}\left(\frac{1}{\mathrm{n}}\sum\:_{\mathrm{i}=1}^{\mathrm{n}}\mathrm{cos}\left(2{\uppi\:}{\mathrm{x}}_{\mathrm{i}}\right)\right)+20+\mathrm{e}$$

This function is widely used to test robustness and global search capability.

## Data Availability

The datasets used and/or analysed during the current study available from the first author on reasonable request.
